# Prevalence of SARS-CoV-2 antibodies among Belgian nursing home residents and staff during the primary COVID-19 vaccination campaign

**DOI:** 10.1080/13814788.2022.2149732

**Published:** 2022-11-28

**Authors:** Eline Meyers, Liselore De Rop, Ellen Deschepper, Els Duysburgh, Tine De Burghgraeve, Pauline Van Ngoc, Marina Digregorio, Simon Delogne, Anja Coen, Nele De Clercq, Laëtitia Buret, Samuel Coenen, An De Sutter, Beatrice Scholtes, Jan Y. Verbakel, Piet Cools, Stefan Heytens

**Affiliations:** a Department of Diagnostic Sciences, Faculty of Medicine and Health Sciences, Ghent University, Ghent, Belgium; b Department of Public Health and Primary Care, EPI-Centre, ACHG, Leuven, Belgium; c Faculty of Medicine and Health Sciences, Biostatistics Unit, Ghent University, Ghent, Belgium; d Department of Epidemiology and Public Health, Sciensano, Brussels, Belgium; e Department of General Medicine, Faculty of Medicine, Research Unit of Primary Care and Health, University of Liège, Liège, Belgium; f Department of Public Health and Primary Care, Faculty of Medicine and Health Sciences, Ghent University, Ghent, Belgium; g Department of Family Medicine and Population Health (FAMPOP) and Vaccine & Infectious Disease Institute (VAXINFECTIO), University of Antwerp, Antwerp, Belgium; h Nuffield Department of Primary Care Health Sciences, University of Oxford, Oxford, UK

**Keywords:** SARS-CoV-2 seroprevalence, nursing homes, nursing home residents, COVID-19 vaccination, vaccination campaign

## Abstract

**Background:**

Nursing home residents (NHR) and staff have been disproportionally affected by the COVID-19 pandemic and were therefore prioritised in the COVID-19 vaccination strategy. However, frail older adults, like NHR, are known to have decreased antibody responses upon vaccination targeting other viral antigens.

**Objectives:**

As real-world data on vaccine responsiveness, we assessed the prevalence of SARS-CoV-2 antibodies among Belgian NHR and staff during the primary COVID-19 vaccination campaign.

**Methods:**

In total, we tested 1629 NHR and 1356 staff across 69 Belgian NHs for the presence of SARS-CoV-2 IgM/IgG antibodies using rapid tests. We collected socio-demographic and COVID-19-related medical data through questionnaires. Sampling occurred between 1 February and 24 March 2021, in a randomly sampled population that received none, one or two BNT162b2 vaccine doses.

**Results:**

We found that during the primary vaccination campaign with 59% of the study population fully vaccinated, 74% had SARS-CoV-2 antibodies. Among fully vaccinated individuals only, fewer residents tested positive for SARS-CoV-2 antibodies (77%) than staff (98%), suggesting an impaired vaccine-induced antibody response in the elderly, with lowest seroprevalences observed among infection naïve residents. COVID-19 vaccination status and previous SARS-CoV-2 infection were predictors for SARS-CoV-2 seropositivity. Alternatively, age ≥ 80 years old, the presence of comorbidities and high care dependency predicted SARS-CoV-2 seronegativity in NHR.

**Conclusion:**

These findings highlight the need for further monitoring of SARS-CoV-2 immunity upon vaccination in the elderly population, as their impaired humoral responses could imply insufficient protection against COVID-19.

**Trial registration:**

This study was retrospectively registered on ClinicalTrials.gov (NCT04738695).

KEY MESSAGES

- In an early response after COVID-19 vaccination, fewer vaccinated nursing home residents (77%) tested positive for SARS-CoV-2 antibodies than staff (98%), suggesting an impaired vaccine-induced antibody response in the elderly.
- In nursing home residents, age ≥ 80 years old, comorbidity and high care-dependency predict SARS-CoV-2 seronegativity.

## Introduction

In Belgium, nursing home residents (NHR) have been disproportionally affected by the COVID-19 pandemic. This resulted in a severe burden on the healthcare system, as, by April 2021, more than half of the total COVID-19 fatal cases in Belgium were NHR [1].

Therefore, the Belgian authorities prioritised nursing home staff (NHS) and NHR in the large-scale COVID-19 vaccination campaign. Between 28 December 2020, and 24 March 2021, all eligible and consenting NHR and NHS were vaccinated with two doses of the BNT162b2 (Pfizer-BioNTech) COVID-19 vaccine [2].

Although, the efficacy of the BNT162b2 vaccine has been thoroughly investigated in clinical trials before market authorisation [3,4], the standardised settings of these trials are not comparable to the reality of vaccine rollout among the general population. Moreover, vaccine-induced antibody responses showed overall favourable results, however, decreased immunogenicity was observed in the older age groups [3]. On top of that, age groups that typically populate NHs in Belgium were vastly underrepresented in the vaccine efficacy trial [5,6]. Yet, it is known that frail older adults can suffer from age-related immune system dysfunction [7].

To document the real-life impact of COVID-19 vaccination on the antibody response of NHR, we assessed the prevalence of SARS-CoV-2 antibodies in NHR from February to December 2021 (the SCOPE study, Supplement 1), and in NHS for comparison. Here, we describe the baseline SARS-CoV-2 prevalence and predictors for seropositivity measured during the primary vaccination campaign in a random sample of NHR and NHS who received none, one or two doses of the BNT162b2 vaccine.

## Methods

### Study design

**Figure 1.**
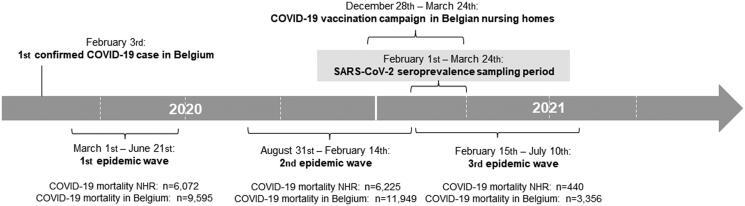
Visual overview of the timing of the baseline sampling period and primary COVID-19 vaccination campaign within the first three SARS-CoV-2 epidemic waves in Belgium. NHR: Nursing home residents. Belgian COVID-19 mortality data during the epidemic waves were retrieved from the Belgian Institute for Health, Sciensano [1,8].

Here, we report a cross-sectional analysis (baseline sampling) of a prospective cohort study that bimonthly assessed the SARS-CoV-2 seroprevalence among NHR and NHS in Belgium over 10 months (the SCOPE study, Supplement 1). The SCOPE study was designed as a national SARS-CoV-2 serosurveillance study. Sample collection started on 1 February 2021. Baseline sampling (day 0, month 0) started staggered so that samples among the different NHs were collected within the first 4 weeks after first sample collection. A study timeline is given in Figure 1.

### Study population

In the SCOPE study, we aimed to recruit 1656 NHR and 1380 NHS across the three regions of Belgium (Flanders, Wallonia and Brussels). Recruitment of the participants occurred between December 2020 and March 2021. A random sample of NHs was selected in proportion to the population and number of NH beds per province. A list of eligible NHR/NHS was used (service flat residents and temporary NHS, employed <1 year after study initiation, or NHS <18 years old were excluded) to randomly select 24 NHR and 20 NHS for participation.

The sample size was calculated assuming a seroprevalence rate of 0.5 at the start of the study and half-width of a 95% confidence interval (CI) of 0.05, a drop-out rate over ten months of 0.2 and 0.4 for NHS and NHR, respectively. Detailed information on the sample size calculation can be found in Supplement 1.

### Ethics

The study was approved by the Ghent University Hospital Ethical Committee (reference number BC-08719) and conducted according to the principles outlined in the Declaration of Helsinki. Each participant signed an informed consent form after being informed about the study’s goal and the sampling procedures. For participants who were incapable of signing the consent form, such as NHR with dementia, consent was given by their legal representative.

### Data collection

#### Antibody testing

We assessed IgM and IgG SARS-CoV-2 antibodies using point-of-care tests (Healgen Scientific LLC., Houston, USA) with a sensitivity of 92.9% and specificity of 96.3% [9]. Testing was performed by trained study personnel.

#### Questionnaire

We asked each participant to complete an online questionnaire (LimeSurvey version 3.22, LimeSurvey GmbH, Hamburg, Germany) on the day of antibody testing. For NHR, the (head) nursing staff completed the questionnaires. In the questionnaire, participant characteristics (e.g. age, sex, job type, care dependency level according to the Katz Index and COVID-19 relevant comorbidities (cardiovascular disease, diabetes, hypertension, immunosuppression, severe renal/lung/cardiac disease, active cancer)) were recorded (Supplementary Table 1) [10,11].

Additionally, participants were asked for their COVID-19 vaccination status, including the type of vaccine (brand name), PCR and/or antigen SARS-CoV-2 tests, COVID-19 confirmation through a CT-scan and the respective test result and date of sampling in case of positive result.

### Statistical analysis

We estimated the SARS-CoV-2 seroprevalence as the number of participants with a positive IgG and/or IgM test divided by the total number of valid test results. The seroprevalence was reported stratified for NHR and NHS, as well as a total seroprevalence with the 95% CI, assessed by a generalised equation estimation (GEE) analysis with independence covariance structure, binomial family and logit link, and robust variance estimator. CIs for seroprevalence were back-transformed from the logit scale.

In addition, the seroprevalence was reported stratified per COVID-19 vaccination status and by history of SARS-CoV-2 infection (either PCR-, antigen- and/or CT-confirmed, self-reported by participants or reported by nursing staff for NHR), averaged over NHs based on a GEE analysis with exchangeable covariance structure.

To assess the associations between seropositivity and age, sex, comorbidities and care dependence level (latter two for residents only), these covariates were included in an adjusted GEE analysis with an exchangeable covariance structure. GEE models only included data subjects for which a valid rapid test result and completed survey was available, to derive the vaccination status and the (self-)reported history of a SARS-CoV-2 infection. All analyses were performed in R, version 4.0.2, using the GEE-library version 4.13–20 and emmeans-library version 1.5.0 (R Core Team, Vienna, Austria).

## Results

### Description of the study cohort

In total, we contacted 108 NHs for participation, of which 33 refused and six dropped out before recruitment of participants, resulting in 69 included NHs. The most common reasons for refusal were the lack of interest in clinical studies or a COVID-19 outbreak at the moment of recruitment. The sampling period was extended by three additional weeks due to the late recruitment of three NHs. An overview of the distribution of the number of NHs and NH population in the study and the total number in Belgium can be found in Supplementary Table 2.

### Participants

#### Participation

In total, 3008 participants were recruited among 69 NHs: 1640 NHR and 1368 NHS; on average, 24 NHR and 20 NHS per NH. Of the total cohort, 2961 (98%) participants were tested for SARS-CoV-2 antibodies and 2840 (94%) participants completed a questionnaire. Combined antibody test results and questionnaire data were available for 2807 (93%) participants (1572 NHR and 1235 NHS).

#### Participant characteristics

Participant characteristics are described in Table 1. The median age of NHR was 87 years old (interquartile range [IQR] 81–91) and 75% was female. Most NHR (69%) suffered from at least one comorbidity and 39% were highly care-dependent (care level C, Cd and D). In comparison, 61% of the NHR cohort was independent to intermediately dependent (care level O, A, B) (Supplementary Table 1).

**Table 1.** Participant characteristics: age, sex, comorbidities, care dependency and job type.

	Total study population *n* = 2840		Residents *n* = 1583		Staff *n* = 1257	
Median age^a^, Interquartile range	74	45;88	87	81;91	43	33;52
Minimum age; maximum age		18;103		46;103		18;71
Male, *n*, %	578	20	394	25	184	15
Female, *n*, %	2262	80	1189	75	1073	85
Comorbidities^b^, *n*, %	1316	46	1086	69	230	18
Cardiovascular disease	694	24	652	41	42	3
Diabetes	318	11	278	18	40	3
Hypertension	686	24	546	34	140	11
Severe heart, lung, and/or renal disease	206	7	180	11	26	2
Immunosuppression	62	2	39	2	23	2
Active cancer	77	3	70	4	7	1
Care dependency, *n*, %						
O^c^	N/A	N/A	159	10	N/A	N/A
A^d^	N/A	N/A	243	15	N/A	N/A
B^e^	N/A	N/A	567	36	N/A	N/A
C^f^	N/A	N/A	215	14	N/A	N/A
Cd^g^	N/A	N/A	370	23	N/A	N/A
D^h^	N/A	N/A	29	2	N/A	N/A
Type of job, *n*, %						
Nursing	N/A	N/A	N/A	N/A	673	54
Administration	N/A	N/A	N/A	N/A	102	8
Paramedic	N/A	N/A	N/A	N/A	168	13
Catering	N/A	N/A	N/A	N/A	105	8
Cleaning	N/A	N/A	N/A	N/A	140	11
Hairdresser/pedicure	N/A	N/A	N/A	N/A	2	0
Other	N/A	N/A	N/A	N/A	67	5

^a^Median age was based on the number of valid questionnaire data points (NH population = 2806; residents = 1572; staff = 1234).

Ages <18 years old for staff and <40 years old for residents were considered invalid and reported as missing. ^b^One or more of the comorbidities below. ^c^Independent, no care needed; ^d^light care needed; ^e^care-dependent; ^f^severe care dependency; ^g^severe care dependency with dementia; ^h^dementia.

The median age for NHS was 43 years (IQR 33–52), 85% was female and 18% suffered from at least 1 comorbidity. More than half of NHS in the cohort were involved in nursing activities (54%), followed by paramedics (e.g. physiotherapists) (13%) and cleaning staff (11%).

### COVID-19 vaccination and infection status

The COVID-19 vaccination and infection status of participants are presented in Table 2. The vaccination coverage among NHR during sampling was slightly higher than NHS. At that time, 68% of NHR was fully vaccinated and 96% had received at least one vaccination dose. For NHS, this was 48% and 85%, respectively. All vaccinated participants were administered a BNT162b2 vaccine, except for three participants (0.02%) (two received AZD1222, AstraZeneca-Oxford, and one mRNA-1273, Moderna vaccine). The mean time between the first and second vaccination was 21 d. For partially vaccinated participants, the mean time between administration of the first dose and antibody testing was 15 d for NHR and 14 d for NHS. For fully vaccinated participants, antibody testing occurred on average 11 and 10 d after administration of the second dose for NHR and NHS, respectively. The spread of the number of days between vaccine administration and SARS-CoV-2 antibody testing is visualised in Supplementary Figure 1. Respectively 38% and 33% of NHR and NHS reported a history of a SARS-CoV-2 infection.

**Table 2.** Vaccination coverage and percentage of (self-)reported history of SARS-CoV-2 infections in Belgian nursing home residents and staff during February–March 2021.

	Not vaccinated		At least one dose^a^		Two doses^a^		(Self-)reported history of infection^b^	
	*n*	%	*n*	%	*n*	%	*n*	%
All (*n* = 2840)	251	9	2589	91	1,684	59	1009	36
Residents (*n* = 1583)	62	4	1521	96	1,077	68	595	38
Staff (*n* = 1257)	189	15	1068	85	607	48	414	33

^a^Participants that had received respectively at least one dose, or two doses (= fully vaccinated) at least one day before the antibody testing date. ^b^(Self-)reported history of infection is defined as a previously reported positive PCR, antigen rapid test and/or CT-scan between February 2020 and the moment of testing.

### SARS-CoV-2 seroprevalence in Belgian NH

Between 1 February and 24 March, 2021, 69% (95% CI: 63–74%) of NHR and 80% (95% CI: 76–83%) of NHS had SARS-CoV-2 antibodies (Table 3). Among all participants, 43% tested positive for IgG antibodies, 1% for IgM antibodies and 29% for both IgM and IgG antibodies (Supplementary Table 3).

**Table 3.** SARS-CoV-2 seroprevalence among residents and staff in Belgian nursing homes during February-March 2021^a^.

	Total study population *n* = 3008		Residents *n* = 1640		Staff *n* = 1368	
	*n* (total)^c^	% (95% CI)	*n* (total)^c^	% (95% CI)	*n* (total)^c^	% (95% CI)
SARS-CoV-2 seroprevalence^b^	2,191 (2,961)	74 (70–78)	1,121 (1,624)	69 (63–74)	1,070 (1,337)	80 (76–83)

^a^Nursing homes were sampled between 1 February and 24 March 2021. The majority of nursing homes (NHs) were sampled during February 2021. Only three NH in the study were sampled during March 2021 due to late recruitment. ^b^95% confidence intervals (CI) were estimated using a generalised estimating equation (GEE) for seropositivity, with an independent covariance structure. ^c^The number between brackets represents the denominator (total number of valid tests) for the specific subgroups.

CI: Confidence interval.

**Figure 2.**
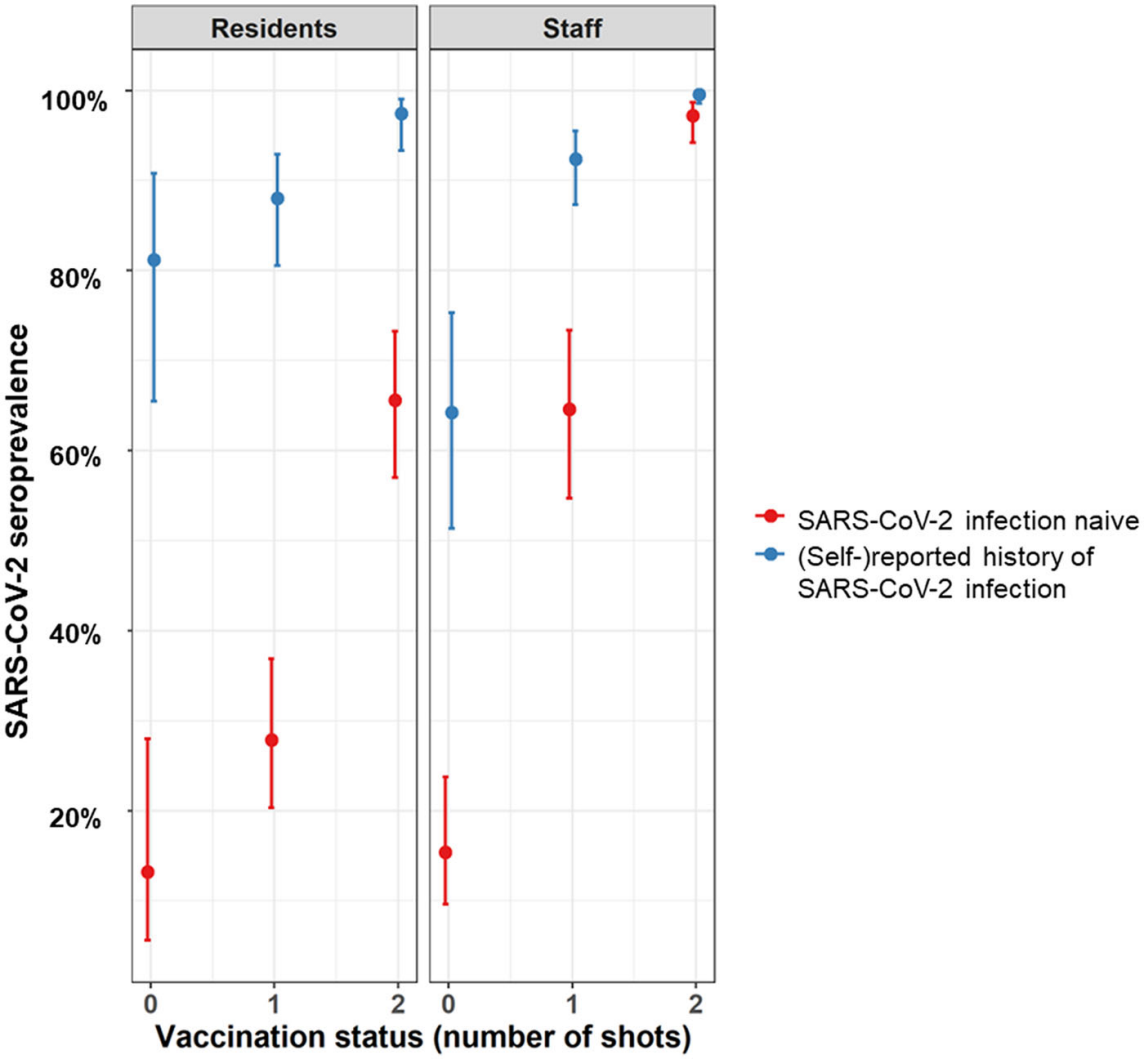
Visualisation of the seroprevalence based on a generalised estimating equation model averaged over nursing homes (with exchangeable structure) by vaccination status (not vaccinated, one dose, two doses) in nursing home residents and staff with and without a (self-) reported history of SARS-CoV-2 infection, Belgium, February–March 2021. Blue dots represent participants with a (self-)reported history of SARS-CoV-2 infection; red dots represent participants without a (self-)reported history of SARS-CoV-2 infection. A (self-)reported history of infection is defined as a previously reported positive PCR-/antigen rapid test- and/or CT-scan COVID-19 test result between February 2020 and the moment of testing.

The SARS-CoV-2 seroprevalences by vaccination status and (self-)reported infection history are presented in Table 4, and visualised in Figure 2. For both partially and fully vaccinated individuals, the SARS-CoV-2 seroprevalence was significantly lower among NHR than NHS (*p* < 0.001). The lowest SARS-CoV-2 seroprevalence among fully vaccinated individuals was observed among infection naive NHR, while among fully vaccinated NHR with a history of SARS-CoV-2 infection, the seroprevalence was comparable to this of NHS of the same group.

**Table 4.** SARS-CoV-2 seroprevalence based on a generalised estimating equation model averaged over nursing homes (with exchangeable structure) by vaccination status (not vaccinated, one dose, two doses) in nursing home residents and staff with and without a (self-)reported history of SARS-CoV-2 infection, Belgium, February–March 2021.

	Nursing home residents	Nursing home staff	
	% (95% CI)		% (95% CI)
Vaccinated two doses^a^	77 (70–83)		98 (96–99)
(Self-)reported history of infection^b^	97 (93–99)		100 (99–100)
No (self-)reported history of infection	66 (57–73)		97 (94–99)
Vaccinated one dose^c^	52 (44–59)		75 (67–81)
(Self-)reported history of infection^b^	88 (81–93)		92 (87–95)
No (self-)reported history of infection	28 (20–37)		65 (55–73)
Not vaccinated	48 (33–63)		37 (29–46)
(Self-)reported history of infection^b^	81 (65–91)		64 (51–75)
No (self-)reported history of infection	13 (6–28)		15 (10–24)

^a^Participants that received the first and second dose of the vaccine dose at least one day before the antibody testing date. ^b^(Self-)reported history of infection is defined as a previously reported positive PCR-/antigen rapid test-/CT-scan COVID-19 test result between February 2020 and the antibody testing date. ^c^Participants that received only the first dose of the vaccine at least one day before the antibody testing date.

### Association between age, (self-)reported history of infection, vaccination status, sex, comorbidities and care dependency on SARS-CoV-2 seropositivity

The associations between age, (self-)reported history of SARS-CoV-2 infection, vaccination status, sex, comorbidities, care dependency level and SARS-CoV-2 seropositivity are presented in Table 5. Both a (self-)reported history of SARS-CoV-2 infection and (partial) COVID-19 vaccination were found to be positively associated with SARS-CoV-2 seropositivity in NHR and NHS (all *p* < 0.001). For NHR only, age ≥ 80 years, the presence of at least one comorbidity and a high care dependency level (category C, Cd, D) were negatively associated with SARS-CoV-2 seropositivity (all *p* < 0.05).

**Table 5.** Association between SARS-CoV-2 seropositivity and age, (self-)reported history of infection^a^, vaccination status, sex, comorbidities and care dependency.

	SARS-CoV-2 seropositivity					
	Residents^b^			Staff^c^		
Determinants	Odds ratio	95% CI	*p* Value	Odds ratio	95% CI	*p* Value
Intercept	5.04	2.75–9.25	<0.001	41.36	19.70–86.82	<0.001
(Self-)reported history of infection^a^	20.31	12.80–32.23	<0.001	8.97	5.64–14.28	<0.001
Partially vaccinated^d^	0.20	0.12–0.32	<0.001	0.05	0.02–0.09	<0.001
Not vaccinated	0.09	0.05–0.18	<0.001	0.00	0.00–0.01	<0.001
Male gender	1.09	0.82–1.45	0.550	0.98	0.55–1.55	0.764
Age 30–39	N/A	N/A	N/A	0.92	0.55–1.55	0.764
Age 40–49	N/A	N/A	N/A	1.60	0.95–2.70	0.079
Age 50+	N/A	N/A	N/A	0.61	0.37–1.03	0.066
Age 70–79	0.60	0.34–1.08	0.090	N/A	N/A	N/A
Age 80–89	0.55	0.33–0.93	0.026	N/A	N/A	N/A
Age 90+	0.46	0.26–0.80	0.006	N/A	N/A	N/A
At least 1 comorbidity^e^	0.70	0.53–0.92	0.011	N/A	N/A	N/A
High care dependency (category C, Cd, D)	0.75	0.58–0.97	0.027	N/A	N/A	N/A

^a^(Self-)reported history of infection is defined as a previously reported positive PCR-/antigen rapid test-/CT-scan COVID-19 test result between February 2020 and the moment of testing. ^b^Adjusted generalised estimating equation model for residents based on reference: fully vaccinated, no (self-)reported history of SARS-CoV-2 infection, no comorbidities, age < 70 years old, female, care level O, A, and B. ^c^Adjusted generalised estimating equation model for staff based on reference: fully vaccinated, no (self-)reported history of SARS-CoV-2 infection, no comorbidities, age < 30 years old, female. ^d^Participants that had received one BNT162b2 dose at least one day before the antibody testing date. ^e^At least one of the following comorbidities: cardiovascular disease, diabetes, hypertension, immunosuppression, severe renal/lung/cardiac disease, and active cancer. CI: confidence Interval. Underlined *p* values are statistically significant.

## Discussion

### Main findings

We measured the prevalence and predictors of SARS-CoV-2 seropositivity among NHR and NHS in Belgium between 1 February and 24 March 2021, during the primary COVID-19 vaccination campaign. A randomly sampled study population of participants that had received one (32%), two (59%), or no vaccine doses (9%) was sampled. During this period, 69% (95% CI: 63–74%) of NHR and 80% (95% CI: 76–83%) of NHS had SARS-CoV-2 antibodies.

Significantly lower seroprevalences were observed among vaccinated NHR than among vaccinated NHS, suggesting that vaccine-induced antibody responses in elderly living in NH were impaired or delayed. More specifically, among both partially and fully vaccinated individuals, the lowest seroprevalences were observed in the group of SARS-CoV-2 infection naive NHR. In contrary, the seroprevalences observed among those fully vaccinated with a history of SARS-CoV-2 infection were similar among NHR and NHS. Among non-vaccinated NHR and NHS (*n*= 244), we found a relatively high seroprevalence of 39% (95% CI: 31–47%), representing the prevalence of infection-induced antibodies. Among NHR, age ≥ 80 years old, comorbidity or high care-dependency were identified as predictors for seronegativity while a (self-)reported history of infection or COVID-19 vaccination predicted seropositivity.

### Comparison with existing literature

Other seroprevalence studies conducted during the COVID-19 vaccination campaign showed similar seroprevalences among other health care workers (HCW). For example, in hospital and primary healthcare workers, a seroprevalence of 64% and 76%, respectively, was observed at the end of February 2021. At that time, there was a similar vaccination coverage as in the NH cohort, with 80% with at least one vaccine dose and 36% fully vaccinated among hospital HCW and 83% with at least one vaccine dose and 66% fully vaccinated among primary HCW [12].

Additionally, our results showed that infection naive NHR have impaired antibody responses upon COVID-19 vaccination, compared to previously infected NHR or NHS. This was also demonstrated in other existing literature. For example, a study in 134 NHR in Spain showed that a previous SARS-CoV-2 infection was associated with higher antibody titres after BNT162b2 vaccination [13]. Another study assessing the antibody concentrations after BNT162b2 vaccination in both NHR and NHS, showed that infection naive NHR had antibody levels that were four times lower than infection naive NHS [14]. A study in Belgium among 40 NHR and 40 NHS vaccinated with BNT162b2, showed similar results, as antibody levels were the lowest among infection naive NHR [15]. Additionally, a comparable study among Belgian NHR showed a decrease in SARS-CoV-2 antibody levels three months after vaccination in infection naive residents. In contrast, in previously infected residents, antibody levels slightly increased [16]. Moreover, the age-related differences observed in antibody responses upon COVID-19 vaccination between elderly and a working-age population have also been described by other studies assessing SARS-CoV-2 antibody levels in community-dwelling older adults [17–19]. Older and frailer individuals had lower chances for seropositivity, which might be explained by immunosenescence, the age-related dysfunction of the immune system. For vaccines targeting other respiratory tract infections, old age and frailty are strongly associated with decreased immune responses upon introduction of newly encountered antigens [20–22]. Moreover, previous evidence indicates that individuals suffering from comorbidities like congestive heart failure and COPD have impaired immune responses upon influenza vaccination [23,24].

In our study, at the moment of testing, Belgium was experiencing the third epidemic wave (started on 15 February 2021). This could explain the high seroprevalence observed in the unvaccinated group, compared to the seroprevalence of 19% and 15% among Flemish NHR and NHS respectively, found in November 2020 in a study of Janssens et al. [25]. In our study, almost half of the unvaccinated participants (54% of NHR and 42% of NHS) reported a history of infection, whereas, in the study by Janssens et al. in November 2020, only 16% of NHR and 15% of NHS reported a previous SARS-CoV-2 infection [25]. However, in our study, the higher prevalence of previous SARS-CoV-2 infection among unvaccinated participants might be biased, as a history of SARS-CoV-2 infection might have been a reason for not getting vaccinated [26,27].

### Strengths and limitations

We assessed the SARS-CoV-2 seroprevalence in a large national sample of NHR and staff, representative for Belgium. However, our study had limitations. Some testing may have occurred too soon after vaccination to detect antibody responses. Moreover, the time between vaccination and antibody testing differed among participants. However, this does not impact the observed differences in seroprevalence between NHR and NHS, as the number of days between vaccine administration and antibody testing was generally the same (Supplementary Figure 1). Additionally, history of SARS-CoV-2 infection was based on (self-)reporting of positive PCR-/antigen-tests/CT-scan. Therefore, the proportion of previously SARS-CoV-2 infected participants could be underestimated due to misreporting, asymptomatic infections and/or lack of routine SARS-CoV-2 testing.

### Implications

Our study findings warrant close monitoring of COVID-19-specific immunity and clinical endpoints among NHR, as they present an impaired humoral immune response upon COVID-19 vaccination. More research is needed to address whether the impaired immune responses also affect COVID-19 disease and mortality outcomes.

## Conclusion

During the primary COVID-19 vaccination campaign in NHs in Belgium (February–March 2021), 69% of NHR and 80% of NHS had SARS-CoV-2 antibodies. These results reflect the positive impact of the vaccination campaign, in addition to pre-existent natural immunity due to the high viral transmission rates in Belgian NH. Our findings show lower seroprevalences among vaccinated NHR compared to vaccinated NHS, suggesting an impaired or delayed vaccine-induced antibody response among elderly living in NH compared to a generally healthy working-age population. This highlights the need for close monitoring of the immunity in this population since the elderly population is at high risk for developing severe cases of COVID-19.

## Supplementary Material

Study Protocol

Table S3

Figure S1

Table S2

Table S1

## Data Availability

The datasets supporting the conclusions of this article are available from the corresponding author on reasonable request.
