# Supplementary material for: Prevalence of SARS-CoV-2 antibodies among Belgian nursing home residents and staff during the primary COVID-19 vaccination campaign
Source: Eur J Gen Pract. 2022 Nov 28;29(2):2149732. doi: 10.1080/13814788.2022.2149732 (PMC10249443; doi:10.1080/13814788.2022.2149732)
Supplement: Study Protocol [file IGEN_A_2149732_SM4492.pdf]

SARS-CoV-2 seroprevalence among nursing home staff and residents in Belgium

Sponsor:

- Sciensano

Principal investigators (PI)

An De Sutter, Department of Public Health and Primary Care, Faculty of Medicine and Health Sciences, Ghent University

Stefan Heytens, Department of Public Health and Primary Care, Faculty of Medicine and Health Sciences, Ghent University

Co-principal Investigator (Co-PI)

Els Duysburgh, Sciensano

Partners:

1. Isabelle Desombere (Sciensano)
2. Piet Cools, Elizaveta Padalko (Department Diagnostic Sciences, Ghent University, Laboratory Clinical Microbiology, University Hospital Ghent)
3. Eline Meyers (Department of Public Health and Primary Care, Ghent University)
4. Ellen Deschepper (Biostatistics Unit, Ghent University)
5. Samuel Coenen, Herman Goossens (University of Antwerp)
6. Jan Verbakel, Ann Van Den Bruel (University of Leuven)
7. Laëtitia Buret, Béatrice Scholtes, Gilles Henrard (University of Liege)
8. Jan de Lepeleire (Crategus, Coordinating and Advising Physicians Flanders)
9. Jean-Francois Moreau (Aframeco, Coordinating and Advising Physicians Wallonia)

Amendment

Protocol V6.2 – June 2021

Table of changes:

Change	Rationale	Affected protocol section
Timepoints of data collection	Seroprevalence is assessed in month 0, 2, 4, 6, 8, 10, instead of week 0, 8, 16, 24, 32 and 40 since data collection within one week was not practically feasible. Bimonthly sample collection of all 69 NHs currently occurs in ± 4 weeks instead of ± 21 days. In each NH, the 2nd, 3rd, 4th, 5th and 6th sample collection are conducted respectively 60, 120, 180, 240 and 300 days after the first sample collection took place in the NHwith a margin of ± 7 days instead of ± 4 days.	2.1 Objectives<br>2.4 Study design and population<br>3. Data analysis<br>4.3 Bias and limitations
Secondary objectives	This study was designed late 2020, when COVID-19 vaccines were still in research development. However, on January 5th, 2021, the vaccination campaign in Belgian NH started. The implementation of vaccines directly impacts the seroprevalence, meaning that some of the secondary objectives were no longer accurate (e.g., assessment of risk factors for infection). Therefore, adjustments were made to the secondary objectives, and tertiary objectives concerning the proportion in the sample that is not vaccinated were added.	2.1 Objectives<br>3. Data analysis
Additional questions concerning vaccine hesitancy	In the first test round we observed that 15% of staff in the sample were not vaccinated against COVID-19. Therefore, we aim to explore the reason behind vaccine hesitancy by adding additional questions in the follow-up questionnaire of the following sampling point concerning if and why they hesitated/are hesitating to get vaccinated as a part of the follow-up questionnaire (one-off).	2.7.2 Demographic and medical data<br>6.3 Appendix: questionnaires
Statistical analysis	The statistical analysis plan was adjusted to the changes made in the secondary objectives and addition of tertiary objectives.	3. Data analysis

1. Background and objectives

Statistics on reverse-transcriptase polymerase chain reaction (RT-PCR)-confirmed SARS-CoV-2 cases and deaths aid in monitoring the dynamics of disease propagation. However, diagnostic RT-PCR testing is far from ideal when trying to estimate the proportion of the population infected, a key indicator for public health decision making in the ongoing COVID-19 pandemic (Roda et al., 2020)[1]. For example, mildly affected or asymptomatic individuals are often not screened. Furthermore, RT-PCR-based testing yields only an epidemiological snapshot, because it fails to detect persons that already cleared SARS-CoV-2, making it difficult to infer the true proportion of the population that has been infected. Also, during the first months of the pandemic, most countries, including Belgium, did not have an adequate RT-PCR testing capacity for screening of anyone suspected or at risk of infection with SARS-CoV-2. As a result, the number of confirmed SARS-CoV-2 infections is largely underestimated (Verity et al., 2020)[2].

In this context, seroprevalence surveys are of utmost importance. These studies can assess the proportion of the population that has already developed SARS-CoV-2 antigen-specific antibodies () and might potentially be protected against subsequent infection (Lipsitch et al., 2020)[3]. As recommended by WHO, monitoring changes of seroprevalence over time is also crucial to anticipate the dynamics of the pandemic and plan an adequate public health response (WHO, 2020)[4,5]. So far, seroprevalence data are available in Belgium for the general population (4.3%; blood donors) (Rode Kruis, 2020)[6], ITG (8.4) (MAY 2020) and hospital staff (6.4%) (Steensels et al., 2020)[7]. Additionally, a national study among health care workers in Belgian hospitals showed an anti-SARS-CoV-2 IgG seroprevalence of 7.7% (Mortgat et al., 2020)[8].

Few data are available concerning the SARS-CoV-2 seroprevalence among nursing home (NH) residents and staff in Belgium, although these populations suffered from severe local SARS-CoV-2 outbreaks. Elderly are at high risk to develop severe cases of COVID-19, as 27% of the

total of the 9878 COVID-19 fatal cases in Belgium (between March 8th and June 2nd 2020) were reported from nursing homes (Sciensano, 2020)[9]. In May 2020, a pilot baseline seroprevalence study was conducted in NHs among Flanders, which showed that 24,5% and 19,1% of respectively NH residents and staff developed SARS-CoV-2 antigen-specific antibodies. Among the nine different nursing homes included in this study, a large variation in seroprevalence was seen ranging from 0,0% to 38,4% and 5,2% to 31,0% for residents and staff respectively (*unpublished data*). However, the degree of seroprevalence and sero-incidence for NHs in Brussels and Wallonia remains unknown. Furthermore, the pilot study conducted in May 2020 assessed the seroprevalence 4 to 6 weeks after the peak incidence of the first epidemic wave. Additionally, when a COVID-19 vaccination will be implemented, it will be of crucial importance to monitor the seroprevalence in Belgian nursing homes and assess the degree of persistance of this vaccine-induced immune response.

Therefore, our major study goals are to assess the current seroprevalence of SARS-CoV-2 in NH residents and staff in Belgium with subsequent 2-monthly follow-up of the sero-incidence over a period of 10 months. Furthermore, many questions remain unsolved about the proportion of asymptomatic cases of SARS-CoV-2 antibody positive NH residents and staff (and about their individual risk factors). Our secondary study goal is to gain additional insights on the association between seropositivity, clinical manifestation and COVID-19 disease outcome.

2. Methods

2.1 Objectives

Primary objectives

1. Assess the seroprevalence of SARS-CoV-2 among NH residents and staff in Belgium at month 0 and at every follow-up visit in the cohort at month 2, 4, 6, 8 and 10..
2. Assess the sero-incidence of SARS-CoV-2 among NH residents and staff in Belgium over five two month time frames and summarised over a ten month time period.

Secondary objectives

1. To assess the seroprevalence according to vaccination status among NH residents and staff in Belgium at month 0 and at every follow-up visit in the cohort at month 2, 4, 6, 8 and 10.
2. To assess the risk of getting a SARS-CoV-2 infection since vaccination between:
   - Residents and staff
   - Seropositive and seronegative participants at all timepoints
   - Individuals with or without previous self-reported SARS-CoV-2 infections
   - Adjusted for the updated vaccination degree of NH at all timepoints
3. To assess the proportion of confirmed COVID-19 deaths and non-COVID-19 related deaths and the time until death since vaccination (within the study timeframe) between sero-positive and -negative participants for NH residents
4. To assess the duration of SARS-CoV-2 specific antibody responses (seroreversion/no antibody response) since vaccination among NH residents and staff in Belgium.

Tertiary objectives

1. To assess the risk of getting SARS-CoV-2 infected between vaccinated and non-vaccinated (at last visit) residents/staff.

2. To assess the proportion of confirmed COVID-19 deaths and non-COVID-19 related deaths and the time until death (within the study timeframe) between vaccinated and non-vaccinated residents.
3. To assess the duration of SARS-CoV-2 specific antibody responses (seroreversion/no antibody response) since self-reported infection in a non-vaccinated residents/staff.

2.2 Project planning

We plan the current proposal into four work packages (WPs) outlined below (Table 1). **WP1** will focus on obtaining ethical clearance. In **WP2** we will take a random sample of NHs, recruit the NHs willing to participate, and subsequently randomly sample all participants. In **WP3**, capillary blood samples and COVID-19 related medical data will be collected over a period of 10 months. Analysis and reporting will be performed in **WP4**. However, given the rapid changes of the epidemic evolution and dynamics, the foreseen timeline is subjected to re-evaluation. If necessary, the foreseen timeline for testing will be re-considered, depending on the epidemiological situation (see special considerations and circumstances).

Table 1. Timelines foreseen in current study proposal. WP, work package. Grey cells indicate months spent for the specific task.

		Nov 2020	Dec 2020	Jan 2021	Feb 2021	March 2021	April 2021	May 2021	June 2021	July 2021	August 2021	Sept 2021	Oct 2021	Nov 2021	Dec 2021	Jan 2022	Feb 2022	March 2022
**WP1**	Ethics																	
**WP2**	Stratified random sampling and recruiting NHs<br>Stratified random sampling and recruitment of staff and residents																	
**WP3**	Capillary blood collection				x		x		x		x		x		x			
**WP4**	Data analysis and reporting																	

2.3 Operational definitions

Study center	One of the partners involved in this study responsible for testing (, Catholic University of Leuven, Ghent University, University of Liege,)
CRC	Clinical Research Coordinator (CRC), person assigned at each study center, responsible for data collection and follow-up of this study.
Contact person	A local NH staff member, assigned as the contact person for communication between the study center and the NH for this specific study.

2.4 Study design and population

We plan to perform a longitudinal cohort study with a 2-month interval serial sampling of 3036, NH residents (1656) and staff (1380), spread over 69 nursing homes throughout Belgium. Sample collection at baseline (day 0, month 0) will start in a staggered way, so all samples among the different nursing homes are collected within the first ± four weeks after collection of the first sample. Follow-up sample collection will occur with a 2-month interval in month 2, month 4, month 6, month 8, month 10 after the baseline collection of that specific nursing home. Ideally, in each NH, the 2nd, 3rd, 4th, 5th and 6th sample collection will take place d 60, 120, 180, 240 and 300 days after the first NH sample collection However, when collection after 60 days is not possible for practical reasons, samples should be collected within a margin of ± 7 days.

The recruited NHs will be equally spread across the Belgian territory according to geographic and demographic characteristics to guarantee general representativity. Forty-four participants (20 staff, 24 residents) will be randomly included per nursing home.

2.4.1 Inclusion and exclusion criteria

NH residents and staff are considered for inclusion in the study. Both residents/staff that did experience COVID-19(-like) symptoms or had a positive COVID-19 test before, as well as residents/staff that did not experience COVID-19(-like) symptoms can participate in this study.

Participants can be included if they are ≥ 18 years old, have been informed (orally and by means of a written information form) on the goal of the study and the procedures of the study and have agreed to participate in the study by means of a signature on the informed consent form. In some cases, NH residents can give oral consent to their legal representative or caregiver, who will confirm this oral consent by their signature. Service flat residents are not considered as target population in this study and are therefore excluded. Temporary staff, employed for a period of less than 1 year starting from baseline collection, will also be excluded. Participants who are unable to provide a capillary blood sample for the point-of-care (POC) test will also be excluded.

2.5 Sample size

To assess the cumulative incidence after 10 months of follow-up in residential care centers in Belgium a sample of 381 participants (residents & staff) is required to estimate a proportion of 0.5 with a half-width of 5%, using a 95% Wilson score CI. As residents/staff are clustered within care facilities, the sample size will be augmented by the design effect. Assuming, 15 participants (7 residents and 8 staff) per institution after 10 months of follow-up, and an ICC of 0.12, the design effect is 2.68 = (1+0.12 x (15-1)). The total sample size required is 1022=381 x 2.68. At least 69 institutions, each including 15 participants, need to be included in this study, which brings the total sample size to 1035 after 10 months follow-up.

In order to obtain at least 15 participants, in 69 institutions after 10 months of follow-up, assuming a drop-out rate of 0.2 and 0.4 for staff (changes in staff, sick leaf and holidays) and residents (deceased, hospitalized or transferred residents), would require 22 sero-negative participants per care facility, (10 staff, 12 residents) in all 69 institutions. Assuming a seroprevalence rate of 0.5 at the baseline moment of the study would require 44 participants per care facility, (20 staff, 24 residents) in all 69 institutions.

A final sample size calculation will be performed to check whether the sample size obtained for the second research question will be sufficient for the analysis of the first research question.

To assess the seroprevalence in residential care centers in Belgium a sample of 381 participants (residents & staff) is required to estimate a seroprevalence of 0.5 with a half-width of 5%, using a 95% Wilson score CI.

As residents/staff are clustered within care facilities, the sample size will be augmented by the design effect. Based on the previous calculation, 44 participants (24 residents and 20 staff) will be sampled per institution. Assuming an ICC of 0.12, the design effect is 6.16 = (1+0.12 x (44-1)). The total sample size required is 2347=381 x 6.16. At least 54 institutions, each including 44 participants, need to be included in this study, which brings the total sample size to 2376 participants.

Therefore, to assess both the first (seroprevalence) and second (cumulative sero-incidence) research question, the sample size will include 69 NHs, each including 44 participants (20 staff, 24 residents). This brings the total sample size up to 3036 participants.

2.6 Sampling procedure

Stratified random sampling of nursing homes.

A stratified random sample of 69 nursing homes from NHs in Belgium will be selected. NH-related features that will be necessary for this method of sampling will be extracted from the existing Sciensano database. The RIZIV number will be necessary to identify the selected nursing homes. The nursing homes will be equally spread over Belgium and randomly sampled within the strata defined by region and province, and in proportion to the population. Random samples from the stratified province will be taken in proportional to the number of beds. The selected NHs will be contacted for agreement to participate in this study by Ghent University.

If a NH refuses to participate, a substitute NH with a comparable profile according to the strata will be contacted. The reason of refusal will be documented to assess possible selection bias.

Stratified random sampling of participants

Nursing homes that have agreed to participate will be asked to make an alphabetic list of the current staff/residents separately (service flat residents and temporary staff excluded) and to send the number of residents and staff to the Ghent University. Staff/residents will be sampled in a 5:6 ratio in each nursing home. Online statistical computing web programming will be used to generate the random sampling schedule out of the staff and resident numbers. The list of randomly selected numbers, including a list with substitute numbers, will be sent back to the NH. The NH's have to sample 20 staff and 24 residents out of their alphabetic list according to delivered random generated numbers.

When a participant refuses (after asking for oral consent) to join the study the subject according to the first following numeric list will be asked to participate. The participants will be assigned to a unique identification code of the following format: CX(X)SXXY or CX(X)RXXY for staff and residents respectively (C, nursing home (centre); S, staff; R, resident). In this way, test results and medical data are pseudonymized for the involved researchers. The link between the identification code and personal data will be safeguarded by the principal investigator. The oral consenting will be confirmed by a means of a signature before or on the day of the first data collection.

2.7 Data Collection

2.7.1 Collection of capillary blood samples and point-of-care test procedures

The capillary blood samples will be obtained using a lancet to finger prick the middle finger and collect a 1-10 drops of capillary blood. A capillary blood drop will be transferred to the

specimen well of a point-of-care (POC) test (COVID-19 IgG/IgM Rapid Test Cassette, Orient Gene Biotech Co.) using a plastic dropper (delivered with COVID-19 IgG/IgM Rapid Test kit). Two drops (ca. 80 µl) of Orient Gene Biotech sample buffer will be immediately added to the buffer well, with avoidance of air bubbles. If the red colour does not move across the test window or if blood is still present in the specimen well after two minutes, one additional drop of sample buffer should be added into the buffer well. The result should be read in 10 minutes, positive test results may be visible as soon as 2 minutes. The result cannot be interpreted after 15 minutes.

The results of the COVID-19 IgG/IgM Rapid Test Cassette should be interpreted as following:

- **Negative**: The coloured line in the control region (C) changes from blue to red. No line appears in the test line region M or G.
- **Anti-COVID-19 IgM positive**: The coloured line in the control region (C) changes from blue to red and a line appears in the test line M.
- **Anti-COVID-19 IgG positive**: The coloured line in the control region (C) changes from blue to red and a line appears in the test line G.
- **Anti-COVID-19 IgG and IgM positive:** The coloured line in the control region (C) changes from blue to red and a line appears in the test line G and M.
- **Invalid test:** Control line is still completely or partially blue and fails to completely change from blue to red. Insufficient specimen sample or incorrect procedural techniques are the most likely reasons for control line failure. The test should be repeated using a new cassette.

For the study any positive result of IgM or IgG will be considered as a positive result. In case of an invalid test, the test is repeated. When it is still invalid it will be noted as such. Additionally, a few drops of capillary blood will be collected on Dried Blood Spot (DBS) collection cards for (semi-)quantitative analysis of vaccine-inudced/infection-induced anti-SARS-CoV-2- specific antibodies as a part of the sub-study performed by Ghent University (details are provided in appendix 6.4).

2.7.2 Demographic and medical data.

Data to be extracted from the Sciensano database are a list of all nursing homes in Belgium with their RIZIV number, postal code, the capacity of each nursing home (number of beds), and suspected (CT and symptoms) and confirmed (PCR and AG POC test) COVID-19 related death rate.

At the NH level, the management will be asked, at baseline, about the proportion of care grade (according to the Katz evaluation scale) among their residents, if a COVID-19 vaccination campaign was organized in the NH yet (date, type of vaccine and vaccination coverage), the suspected and confirmed COVID-19 infection rate, the COVID-19 related and non-COVID-19 related death rate from the beginning of the crisis until now and if and when they had an outbreak (more than one COVID-19 infection that are suspected to be related).With every follow-up sampling timepoint, the NH will be asked about a vaccination campaign (when this did not take place yet at baseline), if COVID-19 testing was performed in their NH, the respective proportion of positive tests and COVID-19 related death rate.

Individual medical data, comprising medical history and if participants have experienced an episode of COVID-19 symptoms or underwent a COVID-19 (PCR/antigen) test (with mention of date and result) or if COVID-19 was confirmed by a CT-scan, reason of missing data of drop outs (death, hospitalisation, sick leave, leaving the job), and whether they received a COVID-19 vaccination (date, type of vaccine, number of doses) in both NH residents and staff will be collected through an online questionnaire at both baseline and follow-up sampling timepoints (LimeSurvey) (See Appendix 6.3 for questionnaires). The CRC will fill out the questionnaire concerning the resident participants during the week of the sampling timepoint. Therefore the head of the nursing staff or the representative of the resident can be asked to provide the data. In this questionnaire, the surveyed medical data will be linked to the participant identification code. Concerning the medical history, the participants (NH staff and residents) will be asked

for COVID-19 relevant preconditions: cardiovascular diseases, diabetes, hypertension, serious heart, lung or renal diseases, immunosuppressing conditions or medication and any form of active cancer. Furthermore, participants (staff and residents) will be asked if they received a COVID-19 vaccination (date, type of vaccine, number of doses), underwent a PCR-/ antigen-test , the date of this test and the result (if more than one test was performed, the date of the first positive test has to be given) or if COVID-19 was confirmed by a CT-scan, and, if they experienced COVID-19 symptoms and when (date). The COVID-19 symptoms episode will be defined according to the Sciensano case definition:

- At least one of following acute principal symptoms not explained by another clear cause: coughing, dyspnoea, chest pain, sudden loss of olfactory sense and/or taste;

OR

- At least two of following symptoms not explained by another clear cause: fever, myalgia, tiredness, rhinitis symptoms, sore throat, head ache, anorexia, diarrhoea, acute confusion , sudden collapse

OR

- Worsening of chronic respiratory symptoms (COPD, asthma, chronic cough) not explained by another clear cause

Additionally, staff members are asked questions concerning vaccine hesitancy (if and why they hesitated to get vaccinated against COVID-19), see Appendix 6.3.

2.7.3 Data collection: organisation

Before start of the study, each study center will be assigned to a selection of NHs that agreed to participate (recruited by Ghent University). Bimonthly, rapid SARS-CoV-2 antibody-testing by means of a point-of-care (POC) test and DBS collection as part of the sub-study conducted by Ghent University (see appendix 6.4) will be organized in each participating NH. The study centers organize the SARS-CoV-2 antibody testing and the data collection in their specifically

assigned NHs. A standard operational procedure (SOP) will be developed to ensure that each study center performs the antibody testing and the data collection in the same standardized way. Additionally, the person(s) from the study centers that will conduct the antibody testing and the data collection in the NH will receive a brief training organized by Ghent University on how to conduct these tasks (this is possible in an online training). Additionally, in each NH, a contact person (local NH staff) will be assigned for communication between the study centers and NHs for study-related inquiries. The interpreted test result of the POC test will be documented by the researchers/CRC with mention of the participant identification code and collection date. If a test was not performed, the reason will be documented (e.g. hospitalisation, sick leave,…). Concerning the reporting of the results of the POC test to individual NH staff members, the test result will be interpreted by the CRC and communicated directly to the individual staff member participant accompanied by a written explanation form about the clinical meaning of the result. Concerning the NH residents, the CRC will communicate the result directly, write it down on a written form explaining the clinical meaning of the result. One copy of this form will be left by the resident, the other copy is meant for the head of the nursing staff, and can be send/forwarded to the Coordinating and advising physician (CRA) , the family or legal representative of the resident and the general practitioner of the resident.

When test results are missing, for instance when a staff member was absent on the sampling day, the study center contacts the NH in order to obtain the test result within 2 days after the sampling day for that NH. At Ghent University, the individual medical data from all participants will be extracted from the LimeSurvey database for statistical analysis. The test results and the questionnaires will both be linked to the unique participant identification number.

3. Data Analysis

3.1 Expected outcome

The benefits to get out of the achievement of this study would be the following*:

- Assessment of seroprevalence at baseline and follow-up in the cohort at month 2, 4, 6, 8, 10 and follow-up of sero-incidence over 5 two month time periods among nursing home staff and residents in NH's in Belgium.

* For every participant, it will be registered whether a COVID-19 vaccine was administered to differentiate between vaccinated and non-vaccinated participants.

3.2 Statistical analysis

Primary objectives

1. <u>To assess the seroprevalence of SARS-CoV-2 among NH residents and staff in Belgium at month 0 and at every follow-up visit in the cohort at month 2, 4, 6, 8 and 10.</u>

The seroprevalence of SARS-CoV-2 among NH residents and staff in Belgium at month 0, and follow-up in the cohort at month 2, month 4, month 6, month 8 and month 10, will be reported stratified for staff and residents, as well as a total seroprevalence with the 95% confidence interval (CI), assessed by a generalized equation estimation (GEE) analysis with independence covariance structure. The average seroprevalence in Belgian NH will be estimated via a generalized equation estimation (GEE) analysis with exchangeable covariance structure. All characteristics will also be displayed for the Belgian regions separately.

2. To assess the sero-incidence of SARS-CoV-2 among NH residents and staff in Belgium over five two month time frames and summarised over a ten month time period.

The sero-incidence of SARS-CoV-2 among NH residents and staff in Belgium over 5 two month time periods, as well as summarized over a period of 10 months, will be estimated as a total sero-incidence, as well as for NH residents staff separately, via a clustered Poisson (or binomial) regression, taking the clustered nature of participants within nursing homes into account.

Secondary objectives

4. To assess the seroprevalence according to vaccination status among NH residents and staff in Belgium at month 0 and at every follow-up visit in the cohort at month 2, 4, 6, 8 and 10.

The seroprevalence among NH residents and staff in Belgium at month 0, and follow-up in the cohort at month 2 (and if relevant at month 4, 6, 8, and 10), will be reported according to the vaccination status (Number of shots: 0, 1 or 2) and for the subgroups 'with' and 'without' a history of self-reported SARS-CoV-2 infection, averaged over NHs based on a GEE analysis with exchangeable covariance structure.

5. To assess the risk of getting a SARS-CoV-2 infection since vaccination between:
    - Residents and staff
    - Seropositive and seronegative participants at all timepoints
    - Individuals with or without previous self-reported SARS-CoV-2 infections
    - Adjusted for the updated vaccination degree of NH at all timepoints

For vaccinated participants only, a survival analysis will be performed to investigate the time (in days) to a SARS-CoV-2 infection since vaccination between sero-positive and -negative

participants at all time points. A participant is considered to have an event when the participant had a positive PCR test result (or a positive antigen rapid test, or a positive CT scan, or a positive saliva test in absence of a PCR test). The survival or censoring time (in days) is registered since 14 days after being fully vaccinated (full dose regimen) until first event (or censoring moment). If the proportional hazard assumption holds, a stratified (by NH) Cox proportional hazards regression model will be used to model the hazard of SARS-CoV-2 infection between sero-positive and -negative participants at all time points (modelled by the inclusion of a time-varying seropositivity status), adjusted for type of participant, history of self-reported SARS-COV-2 infections, and a time-varying NH vaccination degree. The analysis will be performed stratified by type of participant or other variables, if the proportional hazard assumption does not hold.

6. To assess the proportion of confirmed COVID-19 deaths and non-COVID-19 related deaths since vaccination and the time until death (within the study timeframe) between sero-positive and -negative participants for NH residents

For vaccinated NH residents only, a survival analysis will be performed to investigate the proportion of deaths within the study follow-up and the time (in days) to a confirmed COVID-19 death since 14 days after being fully vaccinated (full dose regimen). The survival or censoring time (in days) is registered since14 days after being fully vaccinated until first event (or censoring moment). A non-COVID-19 related death will be considered as a competing risk in the analysis. If the proportional hazard assumption holds, a stratified (by NH) Cox proportional hazards regression model will be used to model the cause-specific hazards between seropositive and seronegative residents. The latter will be included as a time-varying covariate. A history of self-reported SARS-COV-2 infections, a time-varying NH vaccination degree, province, age, gender, care profile, and presence of COVID-specific comorbidities will be

considered for model adjustment.

7. To assess the duration of SARS-CoV-2 specific antibody response (seroreversion/no antibody response) since 14 days after being fully vaccinated among NH residents and staff in Belgium.

The duration of SARS-CoV-2 specific antibody responses among NH residents and staff in Belgium will be modelled by means of an interval-censored survival analysis. Seroreversion will be defined as a negative rapid test result after at least one positive rapid test result or as two succeeding negative test results (implying the participant did not seroconvert after vaccination within two to four months). The time interval to seroreversion/no response is the time interval between 14 days after the (second) vaccination date (full dose regimen) and the first test moment of a negative test result after at least one positive rapid test or of the two succeeding negative rapid test results. As a sensitivity analysis, the event will also be defined as two succeeding negative rapid test results (whether or not after at least one positive rapid test result), if the rapid test result would turn out to be unstable.

Tertiary objectives

Although this study was not designed to assess vaccine effectiveness, end points will be compared between vaccinated and non-vaccinated participants, applying propensity score methods, if sufficient non-vaccinated participants occur in the random selected sample.

8. To assess the risk of getting SARS-CoV-2 infected between vaccinated and non-vaccinated (at last visit) residents/staff.

If a sufficient number of non-vaccinated participants are available in the sample, a survival analysis will be performed to investigate the time (in days) to a SARS-CoV-2 infection

since 14 days after being fully vaccinated between vaccinated and non-vaccinated participants. A participant is considered to have an event when the participant had a reported positive PCR test result (or a positive antigen rapid test, or a positive CT scan, or a positive saliva test in absence of a PCR test) during the baseline or one of the follow-up visits. The survival or censoring time (in days) is registered since 14 days after being fully vaccinated (or since 14 days after main (second) vaccination date in corresponding NH in case of non-vaccinated participants) until first event (or censoring moment).

If the proportional hazard assumption holds, a stratified (by NH) Cox proportional hazards regression model will be used to model the hazard of SARS-CoV-2 infection between staff and residents, and between vaccinated and non-vaccinated participants, adjusting for history of self-reported SARS-COV-2 infections, and a time-varying NH vaccination degree. Propensity score methods will be applied to reduce the effects of confounding. If insufficient non-vaccinated residents would be available in the cohort, the analysis will be performed for staff only.

9. <u>To assess the proportion of confirmed COVID-19 deaths and non-COVID-19 related deaths and the time until death (within the study timeframe) between vaccinated and non-vaccinated residents.</u>

If sufficient non-vaccinated residents would be available, a survival analysis will be performed to investigate the time (in days) to a confirmed COVID-19 death since 14 days after vaccination (or since 14 days after main (second) vaccination date in corresponding NH in case of non-vaccinated participants). A non-COVID-19 related death will be considered as a competing risk in the analysis.

If the proportional hazard assumption holds, a stratified (by NH) Cox proportional hazards regression model will be used to model the cause-specific hazards between vaccinated and

non-vaccinated residents, adjusting for history of self-reported SARS-COV-2 infections, and a time-varying NH vaccination degree. Propensity score methods will be applied to reduce the effects of confounding.

10. To assess the duration of SARS-CoV-2 specific antibody responses (seroreversion/no antibody response) since self-reported infection in a non-vaccinated residents/staff.

If sufficient non-vaccinated participants with self-reported infection would be available in the random selected sample, a survival analysis for interval censored data will be performed to investigate the time (in days) to a SARS-CoV-2 specific seroreversion/no antibody response since self-reported, natural SARS-CoV-2 infection. A participant is considered to have an event (seroreversion/no antibody response) during the interval between a positive and a negative rapid test result during the follow-up visits, or between the between the positive test date and the first visit date in case of a negative rapid test at the first test date. If the rapid test results would turn out to be unstable, a sensitivity analysis will be performed and an event is considered after two succeeding negative rapid test results during the follow-up study visits. The survival or censoring time will be expressed as an interval in days between the positive test date until the visit date with first event (or censoring moment).

A Cox proportional hazards regression model for interval censored data will be used to model the hazard of a SARS-CoV-2 specific seroreversion/no antibody response since natural infection adjusted or stratified for staff and residents.

4. Practical considerations

4.1 Human resources and costs

Personnel costs to manage and deliver the project both in Flanders and Wallonia to perform analysis of the collected data are budgeted for the total amount of 384 273,28 including a full time principal research associate at the university of Ghent and clinical research coordinators at each of the academic centers. The cost for the test material including the Orient Gene tests (10.6 euro per test) and test collecting material (alcohol swabs, lancets, others) is 229 897,80 euro. The costs related to statistical support is estimated at 45 302,40. Other costs (transportation, publication, fee for NH,…) are estimated on 122 322,79. This brings the total up to 781 818,27 euro.

4.2 Quality assurance

As for the questionnaire, this protocol will be reviewed by other researchers and experts in the field. The informed consent and the questionnaire will be available in two languages (French and Dutch). The sample collection and interpretation of the test result will be performed by trained researchers/CRC's. Data analysis will be done by the researchers of Ghent University.

4.3 Bias and limitations

- Loss of follow-up or missing data is possible, for instance, when a NH staff-member is not present at the moment of sample collection or a sample was not collected due to heavy workload. In this case, effort will be made to contact the NH and collect the sample within two days after the sampling day. In case of, for instance hospitalization of NH resident or self-isolation of NH staff, missing test result data is inevitable. In order to prevent missing data in the online questionnaires, results will be monitored and effort will be made to remind participants to fill in the survey (by e-mail/telephonic contact or at the moment of testing).

- Underestimation of the presence of SARS-CoV-19 seroprevalence among this population due to:

- Testing method limitations, however, the COVID-19 IgG/IgM Rapid Test Cassette (Orient Gene Biotech Co.) provides good sensitivity (92,9%) and specificity (96,3%) (Sciensano, 2020).
- Data collection, however, researchers/CRC's in charge of collecting the samples will be trained to ensure correct use of the COVID-19 IgG/IgM Rapid Test Cassette and interpretation of the results to minimize reporting bias.

- Due to practical limitations, seroprevalence data cannot be collected at the same point in time in all participants in this study. Seroprevalence, however, is subjected to fluctuations of the infection rate in the current SARS-CoV-2 pandemic and is therefore also time-dependent. Therefore, the maximal difference between the first and last samples collected within the baseline collection round is set at ± 4 weeks days.

4.4 Distribution of results

The study protocol will be registered at Clinicaltrials.gov (NCT04738695).

Anonymous study results should be made accessible and available as soon as possible after every testing point and at the end of the study to public health authorities involved in management of the COVID-19 epidemic in Belgium. This can be done through a policy brief or presentation. Sciensano will coordinate the distribution of results. These results will be also published on the relevant websites of these institutions. The general public will also be informed regarding these anonymous results through press communications. This will be done by the communication departments of Sciensano and of the study partners.

Scientific peer-reviewed publications (possible short publication, regular paper) will be prepared to add to the body of evidence and availability for the global scientific community and public health decision makers.

4.5 Special considerations and circumstances

Given the uncertainty about the future development of the COVID-19 epidemic, adaptation of testing periods (more frequent, or adding additional testing period(s)and extension of the study might be considered and needed. Additionally, in case of implementation of a SARS-CoV-2 vaccine, it could be appropriate to sample more frequently to estimate the vaccine related sero-incidence.

5. Protection of human subjects

5.1 Risks

The risks for participants are minimal as the capillary blood sampling (fingerprick) is minimally invasive.

5.2 Benefits

Participants will be informed about their test result. In this way, participants will know whether they have developed SARS-CoV-2 specific antibodies, which are possibly protective against new SARS-CoV-2 infections (Lipsitch et al., 2020)[3].

5.3 Confidentiality

Although tests results will be communicated to each participant, at the researchers level, sample results and questionnaires will be pseudonymized via a unique identification code attributed to each participant.

Participant confidentiality will be maintained throughout the investigation. Two different databases will be created. One will contain identifying data such as names, surnames, NH linked

to the participant and a unique registration code assigned to each participant. The other database will contain the study data (POC test results and questionnaire information) and will have as identifier the unique registration code. These 2 databases will be kept separately and will be protected by a password. If the investigator entrusts the data for statistical processing, only the second database will be entrusted to this third person. As such, data in the database for analysis will be pseudonymized. Only the principal investigator will keep the database containing personal information and the unique identification number assigned to each participant.

5.4 Informed consent

Information on the study will be provided by the NH or the CRC/researcher sampling team and informed consent will be obtained from all participants, in their working language. The informed consent (Dutch and French) is attached in respectively Appendix 6.1 and 6.2.

5.5 Ethical committee clearance

The study will be submitted to the Ethical Committee of Ghent University Hospital.

5.6 Study insurance

We have an insurance for this study with Ethias. Insurance reference is: 45.439.317

5.7 Data processing and protection (GDPR)

Data processing and protection for this study complies with the provisions of the regulations for the protection of Personal Data, including but not limited to the EU General Data Protection Regulation 2016/679 of 27 April 2016 and its implementing decrees. Questions and other issues regarding data protection will be addressed to Sciensano Data Protection Officer, e-mail address: dpo@sciensano.be or UZ Ghent Data Protection Officer, e-mail address: dpo@uzgent.be

The General Data Protection Regulation (GDPR, the European legislation governing the protection of personal data, applies to this study. The legal basis on which the data are processed is consent.

- See Article 6 § 1 (a) of the GDPR
- See Article 9 § 2 (a) of the GDPR

6. Appendices

6.1. Appendix 1: informed consent (Dutch)

Informatiebrief voor de deelnemers aan een experiment

Titel van de studie: COVID-19 seroprevalentie in woonzorgcentra

Officiële titel: SARS-CoV-2 seroprevalentie en sero-incidentie bij bewoners en medewerkers uit woonzorgcentra in België.

Beste,

U wordt uitgenodigd om deel te nemen aan een studie. Neem, voor u beslist deel te nemen aan deze studie, voldoende tijd om deze informatiebrief aandachtig te lezen en dit te bespreken met de arts of zijn/haar vertegenwoordiger. Neem ook de tijd om vragen te stellen indien er onduidelijkheden zijn of indien u bijkomende informatie wenst. Dit proces wordt 'informed consent' of 'geïnformeerde toestemming' genoemd. Eens u beslist heeft om deel te nemen aan de studie zal men u vragen om het toestemmingsformulier achteraan te ondertekenen.

WAT IS HET DOEL VAN DE STUDIE?

In opdracht van Sciensano, voert de Vakgroep Volksgezondheid en Eerstelijnszorg en de vakgroep Diagnostische Wetenschappen van de Faculteit Geneeskunde van de Universiteit Gent, in samenwerking met onderzoekers van de Universiteit Antwerpen, KU Leuven en deUniversiteit Luik , een onderzoek uit om na te gaan wie reeds in antistoffen hebt opgebouwd tegen het coronavirus (door natuurlijke infectie/vaccinatie)).
Daarom zal u binnenkort gevraagd worden om getest te worden op de aanwezigheid van COVID-19 specifieke merkers. Daarvoor is het nodig een kleine vingerprik te ondergaan.

De immuunmerkertest leert ons of u reeds antistoffen hebt opgebouwd tegen het SARS-CoV-2, kortweg het coronavirus, Met deze testen zullen we inzicht krijgen in welke mate bewoners en personeel van woonzorgcentra mogelijks beschermd zijn tegen het virus.

Deze studie werd door een onafhankelijke Commissie voor Medische Ethiek verbonden aan het Universitair Ziekenhuis van Gent en de Universiteit Gent vooraf goedgekeurd. De studie wordt uitgevoerd volgens de richtlijnen voor de goede klinische praktijk (ICH/GCP) en de verklaring van Helsinki opgesteld ter bescherming van mensen deelnemend aan klinische studies. In geen geval dient u de goedkeuring door de Commissie voor Medische Ethiek te beschouwen als een aanzet tot deelname aan deze studie.

Deze verzameling van gegevens wordt uitgevoerd onder supervisie van Prof. Dr. An De Sutter en Dr. Stefan Heytens (de hoofdonderzoekers van de Universiteit Gent).

De opdrachtgever van deze studie is het Belgisch instituut voor gezondheid, Sciensano.

WAT HOUDT DEELNAME AAN DE STUDIE IN VOOR U?

Specifiek voor deze studie vragen we u om , gedurende 1 jaar, ca. om de 2 maanden, een vingerprik te ondergaan (maximaal 10 staalafnames in totaal). Dit is een korte prik met een zeer klein en kort naaldje. Na deze prik komen er enkele druppels bloed uit uw vinger, die we met een pipet overbrengen naar een immuunmerker detecterende sneltest en collecteren op een papieren bloedkaartje (Dry Blood Spot). Het resultaat van de sneltest kan na ca. 10 minuten worden afgelezen en u zal dit dezelfde dag nog meegedeeld krijgen. De bloeddruppels op het kaartje worden overgebracht naar het Laboratorium voor Bacteriologisch Onderzoek (Universiteit Gent) voor kwantitatieve analyse van COVID-19 specifieke imuunmerkers in het kader van een sub-studie aan de Universiteit Gent.Bijkomend zal gevraagd worden naar uw medische voorgeschiedenis bij aanvang van de studie en bij elke vingerprik, of u bent getest op het coronavirus (met vermelding van datum en resultaat) en/of u eventuele typische COVID-19 symptomen hebt ervaren de afgelopen periode. Door de huidige ontwikkeling omtrent de implementatie van COVID-19 vaccinatie in de Belgische woonzorgcentra worden bovendien enkele specifieke vragen omtrent de vaccinatie bevraagd.
Deze vragen kunnen ook voor u beantwoord worden door een (hoofd)verpleegkundige.

HOEVEEL DEELNEMERS ZULLEN AAN DEZE STUDIE DEELNEMEN?

Er zullen in totaal 3036 personen aan deze studie deelnemen.

WAT IS DE DUUR VAN DEZE STUDIE?

De verwachte totale duur van de studie voor u is maximaal een vijftiental minuten per vingerprik en het eventuele beantwoorden van de vragen. In totaal loopt deze studie over 1 jaar, waarbij circa om de 2 maanden een vingerprik genomen wordt en een korte vragenlijst afgenomen wordt.

WAT WORDT VERWACHT VAN DE DEELNEMER?

Voor het welslagen van de studie, is het uitermate belangrijk dat u volledig meewerkt met de onderzoeker en dat u zijn/haar instructies nauwlettend opvolgt. U moet bovendien meerderjarig zijn om deel te kunnen nemen aan deze studie. Service flat bewoners behoren niet tot de studiedoelgroep en worden daarom geëxcludeerd. Tijdelijke rusthuismedewerkers, die tewerkgesteld worden voor een periode korter dan

1 jaar na aanvang van de studie worden ook gevraagd niet deel te nemen aan deze studie.

WELKE PROCEDURES VINDEN TIJDENS DE STUDIE PLAATS?

6.1. Procedures:

Het afnemen van de bloeddruppels van de vingertop houdt in dat een arts, verpleegkundige of onderzoeker u een kleine prik zal geven in de vingertop van uw middenvinger. Daarna zullen een aantal druppels bloed op een immuunmerker detecterende sneltest geplaatst worden waarna het resultaat na ca. 10 minuten kan worden afgelezen en verzameld worden op een papieren kaartje voor labo-analyse. De gecollecteerde stalen zullen geregistreerd en, in geval van de bloedkaartjes, bewaard worden in de biobank ‘Scope’. Een biobank is een faciliteit waar menselijk lichaamsmateriaal (zoals bloed, urine, weefselstalen,…) samen met bijkomende gegevens die betrekking hebben tot dit materiaal, worden bewaard. Uw stalen zullen tot het einde van de studie worden bewaard en gebruikt worden om de studiespecifieke analyses op uit te voeren. Na afloop van deze periode, zullen uw stalen vernietigd worden.
De medisch beheerder van deze biobank is Prof. dr. An De Sutter (Contactgegevens medisch beheerder: +32 9 332 61 04, an.desutter@ugent.be). U blijft echter “eigenaar” van uw lichaamsmateriaal. Dat betekent dat u steeds kan eisen dat de biobank uw opgeslagen stalen vernietigt. U moet hiervoor contact opnemen met de onderzoeker, die er dan voor zorgt dat uw opgeslagen lichaamsmateriaal wordt vernietigd. Uw stalen die in het kader van deze studie worden afgenomen en geanalyseerd, zullen steeds gepseudonimiseerd worden na afname.

Voor alle vingerprikken samen - in totaal tien - zal ongeveer 5 ml bloed afgenomen worden voor de studie, dit is ongeveer de inhoud van een koffielepel.
Bij aanvang van de studie zal er gevraagd worden naar uw medische voorgeschiedenis en bij elke vingerprik zal gevraagd worden of u in de afgelopen periode getest werd op COVID-19 (met vermelding van datum en resultaat) en of u COVID-19 gerelateerde symptomen hebt ervaren.

Studieverloop:

Indien u besluit deel te nemen aan de studie en aan alle voorwaarden voor deelname voldoet, zal u onderstaande testen en onderzoeken doorlopen:

U zal, gespreid over een periode van 1 jaar, maximaal 10 keer een vingerprik ondergaan (met steeds ca. 2 maanden tussen twee opeenvolgende vingerprikken). Bij aanvang van de studie zal gevraag worden naar mogelijkse risicofactoren en uw medische voorgeschiedenis en tweemaandelijks of u bent getest op het coronavirus (met vermelding van datum en resultaat) en of u eventuele typische COVID-19 symptomen hebt ervaren de afgelopen periode. Voor woonzorgcentra bewoners zal u voor het beantwoorden van deze vragen geholpen worden door een (hoofd)verpleegkundige.

WAT ZIJN UW RECHTEN BIJ DEELNAME AAN DEZE STUDIE?

De deelname aan deze studie is volledig vrijwillig, er kan op geen enkele manier sprake zijn van dwang. U kunt weigeren om deel te nemen aan de studie en u kunt zich op elk ogenblik terugtrekken uit de studie zonder dat u hiervoor een reden moet opgeven en zonder dat dit op enige wijze een invloed zal hebben op uw behandeling of de verdere relatie met de onderzoeker of de behandelende arts. Dit zal ook geen negatieve invloed hebben op de kwaliteit van de zorgen en uw verdere opvolging.

Uw deelname aan deze studie zal beëindigd worden als de arts meent dat dit in uw belang is. U kan ook voortijdig uit de studie teruggetrokken worden door de onderzoeker als u de in deze informatiebrief beschreven procedures niet goed opvolgt of u de beschreven items niet respecteert.
Indien u uit de studie gehaald wordt, zullen de reeds verzamelde gepseudonimiseerde gegevens in de databank blijven voor analyse, maar er zal geen nieuwe data toegevoegd worden.

Deze studie werd door een onafhankelijke Commissie voor Medische Ethiek verbonden aan het Universitair Ziekenhuis van Gent en de Universiteit Gent. De studie wordt uitgevoerd volgens de richtlijnen voor de goede klinische praktijk (ICH/GCP) en de verklaring van Helsinki opgesteld ter bescherming van mensen deelnemend aan klinische studies. In geen geval dient u de goedkeuring door de Commissie voor Medische Ethiek te beschouwen als een aanzet tot deelname aan deze studie.

Vertrouwelijkheid

In overeenstemming met de Belgische wet van 22 augustus 2002 betreffende de rechten van de patiënt, de Algemene Verordening Gegevensbescherming (of GDPR) (EU) 2016/679 van 27 april 2016 (die vanaf 25 mei 2018 in voege is) en de Belgische wet van 30 juli 2018, betreffende de bescherming van natuurlijke personen in verband met de verwerking van persoonsgegevens en betreffende het vrije verkeer van die gegevens, zal uw persoonlijke levenssfeer worden gerespecteerd en kan u toegang krijgen tot de verzamelde gegevens. Elk onjuist gegeven kan op uw verzoek verbeterd worden.
Uw toestemming om deel te nemen aan de studie betekent dat we gegevens van u verwerken voor het doel van de klinische studie. Deze verwerking van gegevens is wettelijk voorzien op basis van artikel 6, § 1, (b), (e) of (f) en artikel 9, § 2(j) van de Algemene Verordening Gegevensbescherming.

Alle informatie die tijdens deze studie verzameld wordt zal gepseudonimiseerd worden (hierbij kan men uw gegevens nog terugkoppelen naar uw persoonlijk dossier). In het geval van pseudonimisering zal de sleutel tot deze codes enkel toegankelijk zijn voor de onderzoekende arts of de door haar aangestelde vervanger. Enkel de gepseudonimiseerde gegevens zullen gebruikt worden voor analyse van de gegevens en in alle documentatie, rapporten of publicaties (in medische tijdschriften of congressen) over de studie. Vertrouwelijkheid van uw gegevens wordt dus steeds gegarandeerd. Zowel persoonlijke gegevens als gegevens aangaande uw gezondheid zullen verwerkt en bewaard worden gedurende minstens 20 jaar. De verwerkingsverantwoordelijken van de gegevens is de instelling van de hoofdonderzoekers van de studie, maar wordt uitgevoerd door de Universiteit Gent

onder supervisie van Prof. Dr. An De Sutter en Dr. Stefan Heytens (Universiteit Gent). Hun onderzoeksteam zal toegang krijgen tot uw persoonsgegevens. Gegevens uit het patiëntendossier worden verwerkt in het kader van de verbeteringsprocessen van de organisatie en de gezondheidszorg in het algemeen.

De Data Protection Officer kan u desgewenst meer informatie verschaffen over de bescherming van uw persoonsgegevens. Contactgegevens data protection officer UZ Gent: Katya Van Driessche, dpo@uzgent.be. Contactgegevens data protection officer Sciensano: dpo@sciensano.be
Vertegenwoordigers van de opdrachtgever, auditoren, de Commissie voor Medische Ethiek en de bevoegde overheden, allen gebonden door het beroepsgeheim, hebben rechtstreeks toegang tot uw medische dossiers om de procedures van de studie en/of de gegevens te controleren, zonder de vertrouwelijkheid te schenden. Dit kan enkel binnen de grenzen die door de betreffende wetten zijn toegestaan. Door het toestemmingsformulier, na voorafgaande uitleg, te ondertekenen, stemt u in met deze toegang.

De Belgische toezichthoudende instantie die verantwoordelijk is voor het handhaven van de wetgeving inzake gegevensbescherming is bereikbaar via onderstaande contactgegevens:

Gegevensbeschermingsautoriteit (GBA)
Drukpersstraat 35 – 1000 Brussel
Tel. +32 2 274 48 00
e-mail: contact@apd-gba.be
Website: www.gegevensbeschermingsautoriteit.be

Verzekering

De opdrachtgever voorziet in een vergoeding en/of medische behandeling in het geval van schade en/of letsel ten gevolge van deelname aan deze epidemiologische studie. Voor dit doeleinde is een verzekering afgesloten met foutloze aansprakelijkheid conform de wet inzake experimenten op de menselijke persoon van 7 mei 2004 (polisnummer 45.439.317). Indien de arts-onderzoeker van mening is dat er verband met de studie mogelijk is (er is geen verband met de studie bij schade ten gevolge van het natuurlijke verloop van de ziekte of ten gevolge van gekende bijwerkingen van de standaardbehandeling), zal hij/zij de aangifteprocedure bij de verzekering starten. Op dat ogenblik kunnen uw gegevens doorgegeven worden aan de verzekeraar. In het geval van onenigheid met de arts-onderzoeker of met de door de verzekeringsmaatschappij aangestelde expert, en steeds wanneer u dit nodig acht, kunnen u, of in geval van overlijden uw rechthebbenden, de verzekeraar rechtstreeks in België dagvaarden (Ethias Assurance, Rue des Croisiers, 24, 4000 Liège).

WAT ZIJN DE RISICO'S EN VERWACHTE VOORDELEN BIJ DEELNAME AAN DEZE STUDIE?

Deelname aan deze studie brengt voor u waarschijnlijk geen onmiddellijk therapeutisch voordeel. Uw deelname in de studie kan wel helpen om in de toekomst patiënten beter te kunnen helpen.

De waarschijnlijkheid dat u door deelname aan de studie enige schade ondervindt, is zeer laag.

Ook is het mogelijk dat zich andere risico's en ongemakken voordoen die op dit moment nog onbekend zijn. Het is daarom van groot belang om elke nieuwe gezondheidsklacht zo snel mogelijk aan de arts-onderzoeker te melden, ongeacht of de klacht volgens u te maken heeft met de studie of niet.

Wat betreft de vingerprik, zult u een kleine prik voelen. Het bloeden wordt snel gestelpt met behulp van een pleistertje.

U hebt het recht op elk ogenblik vragen te stellen over de mogelijke en/of gekende risico's van deze studie. Mocht u door uw deelname aan de studie toch enig nadeel ondervinden, zal u een gepaste behandeling krijgen.

ZIJN ER KOSTEN VERBONDEN AAN DE DEELNAME AAN DEZE STUDIE?

Uw deelname aan deze studie brengt geen extra kosten mee voor u.

IS EEN VERGOEDING VOORZIEN BIJ DEELNAME AAN DEZE STUDIE?

Er is geen vergoeding voorzien voor deelname aan de studie.

TOT WIE KUNT U ZICH RICHTEN IN HET GEVAL VAN PROBLEMEN OF INDIEN U VRAGEN HEEFT?

Als er een letsel optreedt ten gevolge van de studie, of als u aanvullende informatie wenst over de studie of over uw rechten en plichten, kunt u in de loop van de studie op elk ogenblik contact opnemen met de arts-onderzoeker of de hoofdonderzoeker:

Naam: Prof. Dr. An De Sutter (hoofdonderzoeker)
Adres: C. Heymanslaan 10, 9000 Gent
Telefoonnummer : 09 332 61 04
Email : an.desutter@ugent.be

Naam: Dr. Stefan Heytens (arts-onderzoeker)
Adres: C. Heymanslaan 10, 9000 Gent
Telefoonnummer : 0475 39 58 43
Email : stefan.heytens@ugent.be

TOESTEMMINGSFORMULIER VOOR DE DEELNEMERS AAN EEN EXPERIMENT

Referentienummer van de deelnemer voor deze studie	

Aankruisen door de deelnemer indien akkoord

Ik heb het document "Informatiebrief voor de deelnemers aan een experiment" pagina 1 tot en met 6 gelezen en begrepen en ik heb er een kopij van gekregen. Ik heb uitleg gekregen over de aard, het doel, de duur, de te voorziene effecten van de studie en over wat men van mij verwacht. Ik heb uitleg gekregen over de mogelijke risico's en voordelen van de studie. Men heeft me de gelegenheid en voldoende tijd gegeven om vragen te stellen over de studie en ik heb op al mijn vragen een bevredigend antwoord gekregen, ook op medische vragen.
Ik begrijp dat deelname aan de studie vrijwillig is en dat ik mij op elk ogenblik uit de studie mag terugtrekken zonder een reden voor deze beslissing op te geven en zonder dat dit op enigerlei wijze een invloed zal hebben op mijn verdere behandeling.
Ik begrijp dat auditors, vertegenwoordigers van de opdrachtgever, de Commissie voor Medische Ethiek of bevoegde overheden, mijn gegevens mogelijks willen inspecteren om de verzamelde informatie te controleren. Bovendien ben ik op de hoogte dat bepaalde gegevens doorgegeven worden aan de opdrachtgever van de studie. Te allen tijde zal mijn privacy gerespecteerd worden.
Ik ben me ervan bewust dat deze studie werd goedgekeurd door een onafhankelijke Commissie voor Medische Ethiek verbonden aan het UZ Gent en de Universiteit Gent en dat deze studie zal uitgevoerd worden volgens de richtlijnen voor de goede klinische praktijk (ICH/GCP) en de verklaring van Helsinki, opgesteld ter bescherming van mensen deelnemend aan experimenten. Deze goedkeuring was in geen geval de aanzet om te beslissen om deel te nemen aan deze studie.
Men heeft mij ingelicht dat zowel persoonlijke gegevens als gegevens aangaande mijn gezondheid worden verwerkt en bewaard gedurende minstens 20 jaar. Ik ben op de hoogte dat ik recht heb op toegang en op verbetering van deze gegevens. Aangezien deze gegevens verwerkt worden in het kader van medisch-wetenschappelijke doeleinden, begrijp ik dat de toegang tot mijn gegevens kan uitgesteld worden tot na beëindiging van het onderzoek. Indien ik toegang wil tot mijn gegevens, zal ik mij richten tot de arts-onderzoeker die verantwoordelijk is voor de verwerking ervan.

Ik stem in om deel te nemen aan de volgende delen van de studie:

1) Ik stem ermee in om volledig samen te werken met de arts-onderzoeker. Ik zal hem/haar op de hoogte brengen als ik onverwachte of ongebruikelijke symptomen ervaar.	
2) Ik stem in voor een vingerprik voor de bepaling van COVID-19 immuunmerkers via een sneltest en dit maximaal 10-maal gespreid over een periode van 1 jaar.	
3) Ik stem in met de verzameling van een Dry Blood Spot en dit maximaal 10-maal gespreid over een periode van 1 jaar.	
4) ik stem in met de verzameling van persoonlijke gegevens en gegevens over mijn medische toestand (voorgeschiedenis,COVID-19 testresultaten, typische COVID-19 symptomen)	

Naam en voornaam van de deelnemer	Handtekening	Datum
Naam en voornaam van de arts-onderzoeker*	Handtekening	Datum

2 kopieën dienen te worden vervolledigd. Het origineel wordt door de onderzoeker bewaard in het ziekenhuis gedurende 20 jaar, de kopie wordt aan de deelnemer gegeven.

* Aankruisen door de onderzoeker indien akkoord

Ik verklaar de benodigde informatie inzake deze studie (de aard, het doel, en de te voorziene effecten) mondeling te hebben verstrekt evenals een exemplaar van het informatiedocument aan de deelnemer te hebben verstrekt.	

Ik bevestig dat geen enkele druk op de deelnemer is uitgeoefend om hem/haar te doen toestemmen tot deelname aan de studie en ik ben bereid om op alle eventuele bijkomende vragen te antwoorden.	

Wettelijke vertegenwoordiger:

Relatie met de deelnemer:

Ik verklaar dat men mij heeft geïnformeerd over de vraag om een beslissing te nemen over deelname aan de studie door de persoon die ik vertegenwoordig. Ik handel in zijn/haar beste belang en houd rekening met zijn of haar mogelijke wens. Mijn toestemming is van toepassing op alle punten opgenomen in het toestemmingsformulier voor de deelnemer.	

Naam en voornaam van de wettelijke vertegenwoordiger	Handtekening	Datum

6.1. Appendix 2: informed consent (French)

Feuille d'information pour les participants

Titre de l'étude: Séroprévalence du COVID-19 dans les centres de soins résidentiels

Titre officiel: SARS-CoV-2 séroprévalence et séro-incidence chez résidents et employés dans les centres de soins résidentiels en Belgique.

Cher(e),

Vous êtes invité(e) à participer à une étude. Avant de décider si vous souhaitez participer, il est important que vous compreniez pourquoi cette recherche est réalisée et ce que cela implique pour vous. Un membre de l'équipe investigateur impliqué dans cette étude clinique va parcourir ce document avec vous. Prenez également le temps de poser des questions s'il y a des incertitudes ou si vous avez besoin d'informations supplémentaires. Ce processus s'appelle "consentement éclairé". Quand vous avez décidé(e) de participer à l'étude, il vous sera demandé(e) de signer le formulaire de consentement à la fin.

1. DESCRIPTION ET L'OBJECTIF DE L'ÉTUDE

Réalisé à la demande de Sciensano, le Département des Sciences Diagnostiques et le Département de la Santé Publique et des Soins Primaires de la Faculté de Médecine de l'Université de Gand mène, en collaboration avec des investigateurs de l'Université d'Anvers, de KU Leuven et de l'Université de Liège, une étude pour déterminer qui a déjà construit de l'immunité contre le virus corona (par infection naturelle / vaccination)
Vous recevrez bientôt une invitation d'être testé pour la présence de marqueurs spécifiques du COVID-19. Pour cela, il est nécessaire de subir une petite piqûre au doigt.
Le test des marqueurs immunitaires nous indique si vous avez déjà construit de l'immunité contre le SRAS-CoV-2, bref le coronavirus. Grâce à ces tests, nous saurons dans quelle mesure les résidents et le personnel des centres de soins résidentiels peuvent être protégés contre le virus.

Cette étude a été évaluée par le Comité d'Ethique de l'Hôpital Universitaire de Gand (UZ Gent) et l'Université de Gand, qui a émis un avis favorable. L'étude est menée conformément aux directives des bonnes pratiques cliniques (ICH/GCP) et à la Déclaration d'Helsinki, rédigées pour protéger les personnes qui participent aux études cliniques. En aucun cas, vous ne devez pas considérer l'approbation du Comité d'Ethique médicale comme une incitation à participer à cette étude.

Cette collecte de données est effectuée sous la supervision du Prof. Dr. An De Sutter et Dr. Stefan Heytens (les chercheurs principal de l'Université de Gand)
Le client de cette étude est l'institut belge de santé, Sciensano.

2. AVANTAGES

Spécifiquement pour cette étude, nous vous demandons de subir une piqûre au doigt environ toutes les 2 mois, pendant une période d'une année (maximum 10 échantillons au total). Il s'agit d'une injection courte avec une aiguille très petite et courte. Après cette injection, quelques gouttes de sang sortent de votre doigt, que nous transférons avec une pipette vers un test rapide et vers un papier filtre (Dry Blood Spot) pour détecter des marqueurs immunitaires. Le résultat de ce test rapide peut être lu après environ 10 minutes et vous en serez informé le jour même. Les gouttes de sang sur le papier filtre sont transférées au Laboratoire de recherche bactériologique (Université de Gand) pour l'analyse quantitative des marqueurs immunitaires spécifiques du COVID-19 dans le contexte d'une sous-étude à l'Université de Gand.De plus, on vous posera des questions sur vos antécédents médicaux au début de l'étude et à chaque piqûre de doigt si vous avez été testé pour le coronavirus (avec date et résultat) et / si vous avez vécu des symptômes typiques du COVID-19 dans la période passée. En raison de l'évolution actuelle de la mise en œuvre de la vaccination COVID-19 dans les centres de soins résidentiels belges, certaines questions spécifiques concernant la vaccination sont également remises en question. Une infirmière peut également répondre à ces questions (en cas des résidents).

3. COMBIEN DE PATIENTS PARTICIPERONT À CETTE ÉTUDE?

Au total, 3036 personnes participeront à cette étude.

4. QUELLE EST LA DURÉE DE CETTE ÉTUDE?

La durée totale prévue de l'étude pour vous est au maximum de dix minutes par piqûre. Au total, cette étude se déroulera sur 1 an, avec une piqûre au doigt environ tous les 2 mois. La durée totale prévue de l'étude pour vous est au maximum de quinze minutes par piqûre de doigt et pour répondre aux questions. Au total, cette étude se déroulera sur 1 an, avec une piqûre au doigt environ toutes les 2 mois et un court questionnaire.

5. QU'EST-IL ATTENDU DU PARTICIPANT?

Pour le succès de l'étude, il est extrêmement important que vous coopériez pleinement avec le médecin ou chercheur et que vous suiviez attentivement ses instructions. Vous devez également être majeur pour participer à cette étude.
Les résidents des appartements-services n'appartiennent pas au groupe cible de l'étude et sont donc exclus. Les travailleurs temporaires qui sont employés pendant une période inférieure à 1 an après le début de l'étude sont également invités à ne pas participer à cette étude.

6. QUELLES PROCÉDURES A METTRE EN PLACE PENDANT L'ÉTUDE?

6.1. Procédures:

Prendre les gouttes de sang du bout du doigt signifie qu'un médecin, un personnel infirmier ou un chercheur vous piquera très brièvement dans le bout de votre majeur. Après cela, un certain nombre de gouttes de sang seront placées sur un test rapide pour détecter des marqueurs immunitaires, après quoi le résultat peut être lu après environ 10 minutes et collectées sur un papier filtre pour analyse en laboratoire. Les

échantillons collectés seront enregistrés, et en cas des papiers filtre, garder dans la biobanque ‘Scope’..
Une biobanque est une installation où du matériel corporel humain (tel que du sang, de l'urine, des échantillons de tissus,…) est stocké avec des données supplémentaires relatives à ce matériel. Vos échantillons seront conservés jusqu'à la fin de l'étude et utilisés pour effectuer des analyses spécifiques à l'étude. Passé ce délai, vos échantillons seront détruits.
L'administrateur médical de cette biobanque est le Prof. Dr. An De Sutter (Coordonnées de l'administrateur médical: +32 9 332 61 04, an.desutter@ugent.be). Cependant, vous restez «propriétaire» de votre matière corporelle. Cela signifie que vous pouvez toujours demander à la biobanque de détruire vos échantillons stockés. Vous devez contacter le chercheur pour cela, qui s'assurera alors que vos tissus humains stockés sont détruits. Vos échantillons collectés et analysés dans le cadre de cette étude seront toujours pseudonymisés après leur collecte.

Pour toutes les piqûres au doigt au total, environ 5 ml de sang seront prélevés pour l'étude.
C'est approximativement le contenu d'une cuillère à café
Au début de l'étude, on vous posera des questions sur des facteurs de risques possibles et vos antécédents médicaux et à chaque piqûre au doigt, il vous sera demandé si vous avez été testé pour le COVID-19 au cours de la période précédente (avec la date et le résultat) et si vous avez vécu des symptômes liés au COVID-19 .

6.2. Progression de l'étude:

Si vous décidez de participer à l'étude et remplissez toutes les conditions de participation, vous effectuerez les tests et études suivants:
Réparti sur une période de 1 an, vous subirez une piqûre au doigt au maximum de 10 fois (avec environ 2 mois entre deux piqûres consécutives). Au début de l'étude, on vous demandera vos antécédents médicaux et , bimensuel, si vous avez été testé pour le coronavirus (avec la date et le résultat) et si vous avez présenté des symptômes typiques du COVID-19 au cours de la période écoulée. Pour les résidents, ces questions seront répondues par une infirmière.

7. QUELS SONT VOS DROITS DE PARTICIPER À CETTE ÉTUDE?

La participation à cette étude est totalement volontaire, il ne peut y avoir aucune contrainte. Vous pouvez refuser de participer à l'étude et vous pouvez vous retirer de l'étude à tout moment sans avoir à donner de raison et sans affecter de quelque manière que ce soit votre traitement ou la relation future avec l'investigateur ou le médecin traitant. Cela n'affectera pas non plus la qualité des soins et votre suivi.

Votre participation à cette étude sera interrompue si le médecin le considère dans votre intérêt. Vous pouvez également être retiré prématurément de l'étude par l'investigateur si vous ne suivez pas correctement les procédures décrites dans cette lettre d'information ou si vous ne respectez pas les éléments décrits.

Si vous êtes retiré de l'étude, les données pseudonymisées déjà collectées resteront dans la base de données pour analyse, mais aucune nouvelle donnée ne sera ajoutée.

Cette étude a été pré-approuvée par un Comité Indépendant d'Ethique Médicale affilié à l'Hôpital Universitaire de Gand et à l'Université de Gand. L'étude est menée conformément aux lignes directrices pour les bonnes pratiques cliniques et à la déclaration d'Helsinki préparée pour protéger les personnes participant aux études cliniques. En aucun cas, vous ne devez considérer l'approbation par le comité d'éthique médicale comme une incitation à participer à cette étude.

7.1. CONFIDENTIALITÉ

Conformément à la législation belge du 22 Août 2002 relative aux droits du patient, le Règlement général (UE) n°2016/679 relatif à la protection des données personnelles (ou RGPD) du 27 avril 2016 (en vigueur à compter du 25 mai 2018) et la loi belge du 30 juillet 2018, votre vie privée sera respectée et vous pouvez, si vous le souhaitez, avoir accès aux données collectées. Toute information incorrecte peut être corrigée à votre demande.
Votre consentement à participer à l'étude signifie que nous traitons vos données pour les besoins de l'étude clinique. Ce traitement de données est prévu par la loi sur la base de l'article 6, paragraphes 1, b), e) ou f) et de l'article 9, paragraphe 2 j) du Règlement général sur la protection des données.

Toutes informations collectées au cours de cette étude seront codées (= pseudonymisées) de sorte que vos données peuvent encore être liées au dossier personnel. En cas de pseudonymisation, la clé du code qui vous est assigné ne sera accessible qu'au médecin investigateur/traitant, ou son représentant. Seules les données pseudonymisées seront utilisées dans tout type de documentations, rapports ou publications (dans la littérature scientifique médicale et/ou lors de conférences médicales) concernant cette étude. La confidentialité des données est donc toujours garantie. Les données personnelles et les données concernant votre santé seront traitées et stockées pour une durée d'au moins 20 ans. Le contrôleur des données est l'institution de l'investigateurs principales de l'étude, Prof. Dr. An De Sutter et Dr. Stefan Heytens) (UZ Gand). Son équipe de recherche aura accès à vos données personnelles dans le cadre de cette étude.
Les données du dossier patient sont traitées dans le cadre des processus d'amélioration de l'organisation et des soins de santé en général.

Le Data Protection Officer peut vous fournir plus d'informations sur la protection de vos renseignements personnels. Vous pouvez contacter: Data Protection Officer UZ Gand Katya Van Driessche, dpo@uzgent.be ou Data Protection Officer Sciensano, dpo@sciensano.be

Les représentants du promoteur de l'étude, des auditeurs, le Comité d'éthique médicale et les autorités compétentes, tous liés par le secret professionnel, peuvent accéder directement au dossier médical sous la responsabilité de l'investigateur (ou de l'un de ses collaborateurs) pour contrôler les procédures de l'étude et/ou les données collectées, sans en violer la confidentialité. Ceci n'est possible que dans les

limites des lois concernées. En signant le formulaire de consentement et après avoir reçu les explications préalables, vous consentez également à cet accès.

L'autorité de surveillance belge responsable de l'application de la législation sur la protection des données peut être contactée via les coordonnées suivantes:

Autorité de protection des données (APD)
Rue de la Presse 35 – 1000 Bruxelles
Tel: +32 2 274 48 00
E-mail: contact@apd-gba.be
Site web: www.autoriteprotectiondonnees.be

7.2. ASSURANCES

Le promoteur de l'étude prévoit une compensation et/ou un traitement médical en cas de dommage et/ou dégât causé lors de votre participation à l'étude clinique. Conformément à la législation belge du 7 mai 2004, relative aux expérimentations sur la personne humaine, le promoteur a souscrit un contrat d'assurance « sans fautes » couvrant cette responsabilité (Ethias Assurance, Rue des Croisiers, 24, 4000 Liège, REF. 45.439.317).

8. QUELS SONT LES RISQUES ET LES AVANTAGES ATTENDUS DE LA PARTICIPATION À CETTE ÉTUDE?

Il est peu probable que la participation à cette étude vous apporte un bénéfice thérapeutique immédiat. Votre participation à l'étude peut aider à mieux servir les patients à l'avenir.
La probabilité que vous subissiez un préjudice en participant à l'étude est faible.
Il est également possible que d'autres risques et inconvénients surviennent qui sont encore inconnus à l'heure actuelle. Il est donc très important de signaler tout nouveau problème de santé à l'investigateur le plus tôt possible, que vous pensiez que la plainte est liée ou non à l'étude.

Quant à la piqûre du doigt, vous ressentirez une petite piqûre. Le saignement est rapidement arrêté à l'aide d'un pansement.
Vous avez le droit de poser des questions à tout moment sur les risques possibles et/ou connus de cette étude. Si vous rencontrez un désavantage du fait de votre participation à l'étude, vous recevrez un traitement approprié.

9. Y A-T-IL UN COÛT DE PARTICIPATION À CETTE ÉTUDE?

Votre participation à cette étude n'entraîne aucun coût supplémentaire pour vous.

10. UNE RÉMUNÉRATION POUR LA PARTICIPATION À CETTE ÉTUDE?

Aucune compensation n'est prévue pour la participation à l'étude.

11. QUI POUVEZ-VOUS CONTACTER EN CAS DE PROBLÈMES OU SI VOUS AVEZ DES QUESTIONS?

Si une blessure survient à la suite de l'étude, ou si vous souhaitez des informations supplémentaires sur l'étude ou sur vos droits et obligations, vous pouvez contacter le chercheur ou le chercheur principal à tout moment au cours de l'étude:

Nom: Prof. Dr. An De Sutter (chercheuse principale)
Adresse: C. Heymanslaan 10, 9000 Gand
Numéro de téléphone: 09 332 61 04
Courriel: an.desutter@ugent.be

Nom: Dr. Stefan Heytens
Adresse: C. Heymanslaan 10, 9000 Gand
Numéro de téléphone: 0475 39 58 43
Courriel: stefan.heytens@ugent.be

FORMULAIRE DE CONSENTEMENT POUR LES PARTICIPANTS

Numéro de référence du participant pour cette étude	

J'ai lu et compris le document intitulé « Feuille d'information pour les participants » pages 1 à 5 et j'en ai reçu une copie. J'ai été informé(e) de la nature, du but, de la durée, des effets prévisibles de l'étude et de ce que l'on attend de moi.
Je comprends que la participation à l'étude est volontaire et que je peux me retirer de l'étude à tout moment sans donner de justification de cette décision et sans que cela n'affecte d'une manière ou d'une autre mon traitement ultérieur.
Je suis au courant que cette étude a été approuvée par le comité d'éthique de l'hôpital universitaire de Gand (UZ Gent) et l'université de Gand. L'étude est menée conformément aux directives des bonnes pratiques cliniques (ICH/GCP) et à la Déclaration d'Helsinki, rédigées pour protéger les personnes qui participent aux études cliniques. En aucun cas je ne dois prendre l'avis favorable du comité d'éthique comme une incitation à participer à cette étude.
J'ai été informé que les données personnelles et les données relatives à ma santé sont traitées et stockées pendant au moins 20 ans. Je suis conscient que j'ai le droit d'accéder à cette information et de la corriger. Comme ces données sont traitées à des fins médico-scientifiques, je comprends que l'accès à mes données peut être différé jusqu'à la fin de l'étude. Si je veux accéder à mes données, je m'adresserai au responsable médecin-investigateur.

Cochez la case si vous êtes d'accord

J'accepte de participer aux parties suivantes de l'étude :

1) J'accepte de coopérer pleinement avec l'enquêteur. Je l'informerai si j'éprouve des symptômes inattendus ou inhabituels.	
2) J 'accepte une piqûre au doigt pour la détermination des marqueurs immunitaires COVID-19 via un test rapide et ceci jusqu'à 10 fois sur une période de 1 an.	
3) J'accepte la collection du 'Dry Blood Spot' et ceci jusqu'à 10 fois sur une période de 1 an.	
4) J'accepte la collection des données personnelles et de l'information sur ma situation médicale (antécédents médicaux, des symptômes typique pour la COVID-19, résultats des tests COVID-19)	

Nom et prénom du/de la participant(e)	Signature	Date
Nom et prénom de l'investigateur	Signature	Date

2 copies doivent être complétées. L'original est gardé par l'investigateur à l'hôpital durant une période de 20 ans, la copie est donnée au/à la participant(e).

* A cocher par le médecin si convenu

Je déclare avoir fourni oralement les informations nécessaires sur cette étude (nature, but et effets prévisibles) et fourni une copie du document d'information au participant.	
Je confirme qu'aucune pression n'a été exercée sur le/la participant(e) pour lui permettre de participer à l'étude et je suis prêt(e) à répondre à toute question supplémentaire.	

Représentant légal:

Relation avec le participant:

Je déclare avoir été informé de la demande de décision concernant la participation à l'étude par la personne que je représente. J'agis dans son meilleur intérêt et je prends en compte son éventuel souhait. Mon consentement s'applique à tous les points inclus dans le formulaire de consentement du participant.	

Nom et prénom du représentant légal:	Signature	Date

6.3 Appendix 3: Questionnaires (Dutch/French)

VRAGENLIJSTEN BIJ AANVANG VAN DE STUDIE

Vragenlijst woonzorgcentrum (in te vullen bij aanvang studie)

1. Studiereferentienummer van het woonzorgcentrum

2. Hoe is de verdeling in zorgcategorieën van de bewoners van het woonzorgcentrum (in absolute aantallen)?
   O: A: B: C: Cd: D:
3. Vond er reeds een COVID-vaccinatieronde plaats in het WZC?
   a. 1ste ronde / 2de ronde (datum)
   b. Welk vaccin?
      i. AstraZeneca/Oxford vaccin
      ii. Het BioNtech-Pfizer vaccin
      iii. Het Moderna vaccin
      iv. Andere
2. Hoeveel bewoners werden gevaccineerd (in verhouding tot het totale aantal bewoners?)
3. Hoeveel medewerkers werden gevaccineerd (in verhouding tot het totale aantal medewerkers?)

4. Werd uw WZC getroffen door één of meerdere COVID-19 uitbraken (= meerdere besmettingen in dezelfde periode die – vermoedelijk - aan elkaar gelinkt kunnen worden) vanaf het begin van de crisis tot nu?
   a. In welke periode /perioden werd het WZC getroffen?
   b. Hoeveel personeelsleden en bewoners (bij benadering) werden toen in dezelfde periode positief getest?

5. Hoeveel vermoede of bevestigde COVID-19 besmettingen zijn er binnen het WZC geweest bij bewoners vanaf het begin van de crisis tot nu (bij benadering) (incl. overleden bewoners)?

6. Hoeveel vermoede of bevestigde COVID-19 besmettingen zijn er binnen het WZC geweest bij medewerkers vanaf het begin van de crisis tot nu (bij benadering)

7. Hoeveel bewoners zijn aan COVID-19 overleden vanaf begin crisis tot nu (bij benadering)?
   a. Bevestigde COVID-19 overlijdens
   b. Vermoedelijke COVID-19 overlijdens

8. Hoeveel bewoners zijn aan niet-COVID gerelateerde oorzaken overleden vanaf het begin van de crisis tot nu (bij benadering)

<u>Individuele vragenlijst (in te vullen bij aanvang studie)</u>

Versie voor deelnemende medewerkers:

1. Gelieve uw uniek studienummer in te vullen. (cfr lijst met random cijfers en studienummers, terug gestuurd door het onderzoekscentrum UGent)
2. Gelieve uw leeftijd in te vullen.
3. Wat is uw geslacht? M/V
4. Wat is uw functie (hoofdactiviteit) binnen het WZC
    - Zorg
    - Administratie
    - Paramedisch (kinesitherapeut, ergotherapeut, …)
    - Poetswerk
    - Keuken
    - Kapper/pedicure
    - Andere (vrijwilliger, animatie, …): (*Invulmogelijkheid)*
5. Werkt u op een afdeling voor bewoners met dementie? Ja / neen
6. Lijdt u aan 1 van volgende aandoeningen? Duid aan wat van toepassing is (meerdere antwoordmogelijkheden).
    - Hart- en vaatziekten
    - Diabetes
    - Hoge bloeddruk (hypertensie)
    - Ernstige long-/hart-/nierziekte
    - Verzwakt immuunsysteem (immunosuppressieve aandoeningen) of immuunsysteem verzwakt door langdurig gebruik van immunosuppressieve medicatie (vb. chemotherapie, transplantatiepatiënten, immunosuppressieve reumamedicatie, corticoïden…)
    - Actieve kankeraandoening
    - Geen van bovenstaande

- Bent u gevaccineerd tegen COVID-19?
    - Ja, 1 dosis
    - Ja, 2 dosissen
    - Neen

    - Zo ja, welk vaccin kreeg u toegediend
        - Het AstraZeneca/Oxford vaccin
        - Het BioNtech-Pfizer vaccin
        - Het Moderna vaccin
        - andere
    - Zo ja, datum van vaccinatie (eerste spuit).

7. Bent u dit seizoen gevaccineerd tegen griep? *(Deze vraag zal terugkomen in de vragenlijst bij laatste follow-up)*
8. Bent u ooit getest geweest op het coronavirus (via PCR/ag-sneltest/of radiologisch via CT-scan)? Ja/Nee

9. Zo ja, Bent u ooit **positief** getest geweest op het coronavirus? (Meerdere antwoordmogelijkheden)
    - Ja, via PCR-test (test waarbij men via een neuswisser een staal afneemt en u na labo-analyse het resultaat krijgt)
    - Ja, via antigen sneltest (test waarbij men via neuswisser een staal afneemt en men binnen het kwartier het resultaat kan aflezen)
    - Ja, radiologisch, via een CT-scan
    - neen

9. Zo ja, datum van 1ste positieve PCR-test of sneltest (bij benadering)?
10. Indien COVID-19 radiologisch bevestigd werd via een CT-scan , vermeld de datum van deze CT-scan.
11. Indien u ooit positief getest bent , waar lag de vermoedelijke bron van besmetting ?
    - Binnen het WZC
    - Buiten het WZC
    - De bron van de besmetting was mij onbekend
12. Hebt u tussen februari 2020 en nu een periode 1 of meerdere van volgende symptomen ervaren, zonder dat hier een duidelijke verklaring voor was?
    Duid aan wat van toepassing is (meerdere antwoordmogelijkheden).
    - Hoest
    - Kortademigheid
    - Pijn op de borst
    - Smaakverlies
    - Geurverlies
    - Verergering van chronische respiratoire aandoeningen (vb. COPD, astma, chronische hoest,…)
    - Koorts
    - Spierpijn
    - Vermoeidheid
    - Loopneus/verstopte neus
    - Keelpijn
    - Hoofdpijn
    - Verlies van eetlust
    - Diarree
    - Acute verwardheid
    - Plotse val
13. Zo ja, wanneer hebt u, bij benadering, deze symptomen ervaren? Gelieve, bij benadering, de dag aan te geven waarop deze symptomen zijn begonnen.

Versie voor deelnemende bewoners

(in te vullen door de onderzoeker; bevragen bij de (hoofd)verpleegkundige of de vertegenwoordiger van de bewoner)

Beste bewoner, beste vertegenwoordiger van de bewoner

1. Gelieve het uniek studienummer van de deelnemer in te vullen. (cfr lijst met random cijfers en studienummers, terug gestuurd door het onderzoekscentrum UGent)
2. Gelieve de leeftijd van de deelnemer in te vullen.
3. Wat is het geslacht van de deelnemer? M/V
4. Tot welke zorgcategorie behoort de bewoner?
   O: A: B: Cc: Cd: D:

5. Verblijft de deelnemer in een afdeling voor bewoners met dementie?
6. Lijdt de deelnemer aan 1 van volgende aandoeningen? Duid aan wat van toepassing is (meerdere antwoordmogelijkheden).
   - Hart- en vaatziekten
   - Diabetes
   - Hoge bloeddruk (hypertensie)
   - Ernstige long-/hart-/nierziekte
   - Verzwakt immuunsysteem (immunosuppressieve aandoeningen) of immuunsysteem verzwakt door langdurig gebruik van immunosuppressieve medicatie (vb. chemotherapie, transplantatiepatiënten, immunosuppressieve reumamedicatie,…)
   - Actieve kankeraandoening
   - Geen van bovenstaande
7. Is de deelnemer gevaccineerd tegen COVID-19?

   - Ja, 1 dosis
   - Ja, 2 dosissen
   - Neen

     - Zo ja, welk vaccin kreeg de deelnemer toegediend
       - Het AstraZeneca/Oxford vaccin
       - Het BioNtech-Pfizer vaccin
       - Het Moderna vaccin
     - Zo ja, datum van vaccinatie (eerste spuit).
8.
9. Is de deelnemer ooit getest geweest op het coronavirus (via PCR/ag-sneltest/of radiologisch via CT-scan)? Ja/Nee
10. Zo, ja. Is de deelnemer ooit positief getest geweest op het coronavirus?
    - Ja, via PCR-test (test waarbij men via een neuswisser een staal afneemt en u na labo-analyse het resultaat krijgt)

    - Ja, via antigen sneltest (test waarbij men via neuswisser een staal afneemt en men binnen het kwartier het resultaat kan aflezen)

    - Ja, radiologisch, via een CT-scan

- Neen

11. Zo ja, datum van 1[ste] positieve PCR-test of sneltest (bij benadering)?
12. Indien COVID-19 radiologisch bevestigd werd via een CT-scan , vermeld de datum van deze CT-scan.

13. Heeft de deelnemer tussen februari 2020 en nu een periode 1 of meerdere van volgende symptomen ervaren, zonder dat hier een duidelijke verklaring voor was? Duid aan wat van toepassing is (meerdere antwoordmogelijkheden).

- Hoest
- Kortademigheid
- Pijn op de borst
- Smaakverlies
- Geurverlies
- Verergering van chronische respiratoire aandoeningen (vb. COPD, astma, chronische hoest,…)
- Koorts
- Spierpijn
- Vermoeidheid
- Loopneus/verstopte neus
- Keelpijn
- Hoofdpijn
- Verlies van eetlust
- Diarree
- Acute verwardheid
- Plotse val

14. Zo ja, wanneer heeft de deelnemer, bij benadering, deze symptomen ervaren? Gelieve de dag aan te geven waarop deze symptomen zijn begonnen.

(in te vullen 2-maandelijks in de week dat de test afgenomen wordt)

1. Studiereferentienummer van het woonzorgcentrum.

Vraag 2, 3 en 4 worden enkel toegevoegd indien bij de vorige vragenlijst neen werd geantwoord.

2. Vond er reeds een COVID-vaccinatieronde plaats in het WZC?

2.3. 1ste ronde / 2de ronde (datum)

2.4. Welk vaccin?

2.4.2. AstraZeneca/Oxford vaccin

2.4.3. Het BioNtech-Pfizer vaccin

2.4.4. Het Moderna vaccin

2.4.5. Andere

3. Hoeveel bewoners werden gevaccineerd (in verhouding tot het totale aantal bewoners?)
4. Hoeveel medewerkers werden gevaccineerd (in verhouding tot het totale aantal medewerkers?)

5. Hebt u bewoners/medewerkers georganiseerd laten testen op COVID-19 in de afgelopen 2 maanden (tussen vorige testronde en nu)?
6. Zo ja,
    - Via PCR-testing, hoeveel?
    - Via antigen sneltesten, hoeveel?

7. Zo ja, wie heeft u laten testen? Duid aan wat van toepassing is.
    - Bewoners/medewerkers met symptomen
    - Een afdeling/gang (preventief vb. na risicocontact)
    - Alle bewoners/medewerkers
    - Alleen medewerkers
    - Alleen bewoners
    - Andere combinaties?: *(open tekstveld)*

8. Zo ja, hoeveel bewoners/medewerkers testten positief?

    Voor de PCR test:

    Voor de sneltest:

9. Hoeveel bewoners zijn de afgelopen 2 maanden overleden aan COVID-19?
    - Aantal bevestigde COVID-19 overlijdens
    - Aantal vermoedelijke COVID-19 overlijdens

Individuele vragenlijst

<u>(in te vullen 2-maandelijks in de week dat de test afgenomen wordt)</u>

Versie voor deelnemende medewerkers:

1. Gelieve uw uniek studienummer in te vullen.
2. Neemt u nog deel aan de studie (zal deze vragenlijst vervolledigd worden) ?
   Zo neen, waarom niet?

Door de onderzoeker in te vullen

   a. Tussentijdse afwezigheid in de studie (ziekte, vakantie, hospitalisatie …)
   b. Deelnemer wenst niet meer deel te nemen
   a. Vermoedelijk of bewezen COVID-19 overlijden datum van overlijden:
   b. Niet-COVID-19 gerelateerd overlijden datum van overlijden:
   c. Medewerker is niet langer werkzaam in het WZC

Vraag 3 wordt enkel toegevoegd indien voordien neen werd geantwoord.

3. Bent u gevaccineerd tegen COVID-19?
   a. Ja, 1 dosis
   b. Ja, 2 dosissen
   c. Neen
   d. Zo ja, welk vaccin kreeg u toegediend
      i. Het AstraZeneca/Oxford vaccin
      ii. Het BioNtech-Pfizer vaccin
      iii. Het Moderna vaccin
      iv. andere
   e. Zo ja, datum van vaccinatie (eerste spuit).

4. Bent u in de afgelopen twee maanden getest geweest op het coronavirus (via PCR/ag-sneltest/of radiologisch via CT-scan)? Ja/Nee

5. Zo ja. Bent u in de afgelopen 2 maanden (tussen de vorige testronde en nu) positief getest?
   Meerdere antwoordmogelijkheden
   - Ja, via PCR-test (test waarbij men via een neuswisser een staal afneemt en u na labo-analyse het resultaat krijgt)
   - Ja, via antigen sneltest (test waarbij men via neuswisser een staal afneemt en men binnen het kwartier het resultaat kan aflezen)
   - Ja radiologisch, via een CT-scan
   - neen

6. Zo ja, datum van 1ste positieve PCR-test of sneltest in de afgelopen 2 maanden (bij benadering)?

7. Indien COVID-19 radiologisch bevestigd werd via een CT-scan , vermeld de datum van deze CT-scan.
8. Hebt u in de afgelopen 2 maand (tussen de vorige testronde en nu) een periode 1 of meerdere van volgende symptomen ervaren, zonder dat hier een duidelijke verklaring voor was? Duid aan wat van toepassing is (meerdere antwoordmogelijkheden).
   - Hoest
   - Kortademigheid
   - Pijn op de borst
   - Smaakverlies
   - Geurverlies
   - Verergering van chronische respiratoire aandoeningen (vb. COPD, astma, chronische hoest,…)
   - Koorts
   - Spierpijn
   - Vermoeidheid
   - Loopneus/verstopte neus
   - Keelpijn
   - Hoofdpijn
   - Verlies van eetlust
   - Diarree
   - Acute verwardheid
   - Plotse val

9. Zo ja, wanneer hebt u, bij benadering, deze symptomen ervaren? Gelieve de dag aan te geven waarop deze symptomen zijn begonnen.

10. Indien van toepassing, bent u gevaccineerd met een vaccin tegen COVID-19?
    Zo ja, datum van de uw vaccinatie. *(Deze vraag zal ingevoerd worden zodra van toepassing)*

***Vaccine hesitancy* bij medewerkers van woonzorgcentra in België*:* éénmalige vragenlijst als onderdeel van de follow-up vragenlijst**

A . Bent u reeds gevaccineerd tegen COVID-19?

- Ja, ik ben reeds gevaccineerd (1 of 2 dosissen)
  - Heeft u getwijfeld voor u hebt besloten zich te laten vaccineren? Ja/nee
    - Indien ja → vraag B + C
    - Indien nee → einde vragenlijst
- Ik word binnenkort gevaccineerd
  - Heeft u getwijfeld om zich te laten vaccineren? Ja/nee
    - Indien ja → vraag B + C
    - Indien nee → vraag D
- Ik twijfel of ik me zou laten vaccineren
  - Vraag B
- Ik heb besloten om me niet te laten vaccineren
  - Vraag E

B . Wat is de hoofdreden waarom u twijfelt/twijfelde om gevaccineerd te worden tegen COVID-19?

- Ik ben/was bezorgd over onbekende toekomstige effecten van het vaccin
- Ik ben/was bezorgd over de bijwerkingen van het vaccin
- Ik vertrouw/vertrouwde vaccinaties niet.
- Ik heb/had het idée dat het onwaarschijnlijk is dat ik ernstig ziek zal worden van het coronavirus
- Ik vind/vond dat de impact van het coronavirus wordt overdreven
- Ik denk/dacht dat het vaccin niet kan verhinderen dat ik besmet zal geraken met het virus
- Ik heb een aandoening waardoor het vaccin niet veilig is voor mij
- Ik ben reeds besmet geweest met COVID-19 dus ik heb geen vaccin nodig
- Ik ben bang van naalden
- Ik heb geen mensen in mijn omgeving die tot de risicogroep behoren
- Anders : _____ (deelnemers krijgen een invulveld)
- Ik verkies niet te antwoorden

C. Wat is de hoofreden waarom u besloot u toch te laten vaccineren?

- Ik vond de resultaten van de eerste vaccinaties geruststellend
- Ik had meer tijd nodig om me te informeren
- Een vriend/ familielid/ gezondheidsporfessional heeft het mij aanbevolen
- Iemand uit mijn omgeving werd enrstig ziek van het coronavirus
- Ik voelde de nood om te laten vaccineren om andere kwetsbare mensen te beschermen tegen het virus
- Ik ben bang dat ik zonder vaccinatie niet meer zal kunnen reizen
- Ik denk dat het vaccin deze gezondheidscrisis zal oplossen
- Ik voelde me onder druk gezet door mijn professionele omgeving
- Ik voelde me onder druk gezet door familieleden/vrienden
- Ik volgde de overheidsaanbeveling om me te laten vaccineren (veiligheidsraad, sensibilisering affiches, etc.)
- Ik ben tijdens de crisis een individu met risico op COVID-19 geworden
- Ik werd geïnformeerd over het risico van ernstig ziek te worden op COVID-19
- Ik werd me bewust van het voorkomen van nieuwe, meer besmettelijke varianten van het coronavirus
- Anders : _____ (deelnemers krijgen een invulveld)
- Ik verkies niet te antwoorden

D . Wat is/zijn de redenen waarom u u later laat vaccineren? *(meerdere antwoordmogelijkheden)*

- Ik was afwezig tijdens de eerste vaccinatieronde in het WZC (ziekte/vakantie) en heb sindsdien niet meer de kans gehad om me te laten vaccineren
- Ik heb een chronische aandoening en vaccinatie werd op het moment van de eerste vaccinatieronde niet aanbevolen
- Ik was zwanger en en vaccinatie werd op het moment van de eerste vaccinatieronde niet aanbevolen

- Ik wens in de nabije toekomst zwanger te worden
- Ik ben aan het wachten op een specifiek vaccin
- Anders : ___ (deelnemers krijgen een invulveld)
- Ik verkies niet te antwoorden

E . Wat is de hoofdreden waarom u niet gevaccineerd wenst te worden tegen COVID-19?

- Ik ben bezorgd over onbekende toekomstige effecten van het vaccin
- Ik ben bezorgd over de bijwerkingen van het vaccin
- Ik vertrouw vaccins niet.
- Ik denk dat het onwaarschijnlijk is dat ik ernstig ziek zal worden van het coronavirus
- Ik vind dat de impact van het coronavirus wordt overdreven
- Ik denk dat het vaccin niet kan verhinderen dat ik besmet zal geraken met het virus
- Ik heb een aandoening waardoor het vaccin niet veilig is voor mij
- Ik ben reeds besmet geweest met COVID-19 dus ik heb geen vaccin nodig
- Ik ben bang van naalden
- Ik heb geen mensen in mijn omgeving die tot de risicogroep behoren
- Anders : _____ (*deelnemers krijgen een invulveld*)
- Ik verkies niet te antwoorden

Versie voor deelnemende bewoners

(in te vullen door de onderzoeker; bevragen bij de (hoofd)verpleegkundige of de vertegenwoordiger van de bewoner):

1. Gelieve het uniek studie nummer van de deelnemer in te vullen.
2. Neemt de bewoner nog deel aan de studie (zal deze vragenlijst vervolledigd worden)?
   ja/neen
   Zo neen, waarom niet?
   a. Vermoedelijk of bewezen COVID-overlijden (datum overlijden)
   b. Niet-COVID-19 gerelateerd overlijden (datum overlijden)
   c. Bewoner wenst niet meer deel te nemen

Vraag 3 wordt enkel toegevoegd indien voordien neen werd geantwoord.

3. Is bewoner gevaccineerd tegen COVID-19?
   a. Ja, 1 dosis
   b. Ja, 2 dosissen
   c. Neen
   d. Zo ja, welk vaccin werd toegediend
      i. Het AstraZeneca/Oxford vaccin
      ii. Het BioNtech-Pfizer vaccin
      iii. Het Moderna vaccin
      iv. andere
   e. Zo ja, datum van vaccinatie (eerste spuit).
4.

5. Is de deelnemer in de afgelopen twee maanden getest geweest op het coronavirus (via PCR/ag-sneltest/of radiologisch via CT-scan)? Ja/Nee
6. Zo, ja. Is de deelnemer in de afgelopen 2 maand (tussen de vorige testronde en nu) **positief** getest op het coronavirus?
   - Ja, via PCR-test (test waarbij men via een neuswisser een staal afneemt en u na labo-analyse het resultaat krijgt)
   - Ja, via antigen sneltest (test waarbij men via neuswisser een staal afneemt en men binnen het kwartier het resultaat kan aflezen)
   - Ja, radiologisch, via een CT-scan
   - Neen

7. Zo ja, datum van 1ste positieve PCR-test of sneltest de voorbije 2 maanden (bij benadering)?

8. Indien de COVID-19 radiologisch bevestigd werd via een CT-scan, vermeld de datum waarop dit is gebeurd.

9. Heeft de deelnemer in de afgelopen 2 maand (tussen de vorige testronde en nu) een periode 1 of meerdere van volgende symptomen ervaren, zonder dat hier een duidelijke verklaring voor was? Duid aan wat van toepassing is (meerdere antwoordmogelijkheden).
   - Hoest
   - Kortademigheid
   - Pijn op de borst
   - Smaakverlies
   - Geurverlies
   - Verergering van chronische respiratoire aandoeningen (vb. COPD, astma, chronische hoest,…)
   - Koorts
   - Spierpijn
   - Vermoeidheid
   - Loopneus/verstopte neus
   - Keelpijn
   - Hoofdpijn
   - Verlies van eetlust
   - Diarree
   - Acute verwardheid
   - Plotse val

10. Zo ja, wanneer heeft de deelnemer, bij benadering, deze symptomen ervaren? Gelieve de dag aan te geven waarop deze symptomen zijn begonnen.

11. Indien van toepassing, is de deelnemer gevaccineerd met een vaccin tegen COVID-19? Zo ja, datum van de vaccinatie *(Deze vraag zal ingevoerd worden zodra van toepassing)*

Questionnaire sur les foyers de soins (à remplir au début de l'étude)

9. Numéro de référence de l'étude du centre d'hébergement

10. Quelle est la répartition en catégories de soins des résidents du centre d'hébergement (en chiffres absolus)?

    O: A: B: C: CD: RÉ:

    11. Une campagne de vaccination COVID a-t-elle déjà eu lieu au WZC?
    1er tour / 2ème tour (date)
    b. Quel vaccin?
    je. Vaccin AstraZeneca / Oxford
    ii. Le vaccin BioNtech-Pfizer
    iii. Le vaccin Moderna
    iv. Autres

    2. Combien de résidents ont été vaccinés (par rapport au nombre total de résidents?)
    3. Combien d'employés ont été vaccinés (par rapport au nombre total d'employés?)

11. Votre hôpital a-t-il été touché par une ou plusieurs flambées de COVID-19 (= plusieurs infections au cours de la même période qui - vraisemblablement - peuvent être liées les unes aux autres) depuis le début de la crise jusqu'à maintenant?
    a. Au cours de quelle (s) période (s) le WZC a-t-il été affecté?
    b. Combien de membres du personnel et de résidents (environ) ont été testés positifs au cours de la même période?

12. Combien d'infections au COVID-19 suspectées ou confirmées y a-t-il eu dans le WZC parmi les résidents depuis le début de la crise jusqu'à aujourd'hui (environ) (y compris les résidents décédés)?

13. Combien d'infections au COVID-19 suspectées ou confirmées ont été commises au sein du WZC parmi les employés depuis le début de la crise jusqu'à maintenant (approximatif)

14. Combien de résidents sont morts du COVID-19 depuis le début de la crise jusqu'à maintenant (environ)?
    a. Décès confirmés au COVID-19
    b. Décès suspectés de COVID-19

15. Combien de résidents sont décédés de causes non liées au COVID depuis le début de la crise jusqu'à aujourd'hui (approximatif)

Questionnaire individuel (à remplir au début de l'étude)

Version pour les employés participants:

10. Veuillez saisir votre numéro d'étudiant unique. (liste cfr avec nombres aléatoires et numéros d'étude, retournée par le centre de recherche UGent)
11. Veuillez entrer votre âge.
12. Quel est ton sexe? H / F
13. Quelle est votre position (activité principale) au sein du WZC
    - Se soucier
    - Administration
    - Paramédical (kinésithérapeute, ergothérapeute, ...)
    - Nettoyage
    - cuisine
    - Coiffeur / pédicure
    - Autre (bénévole, animation, ...): (Remplissez l'option)
14. Travaillez-vous dans un service pour résidents atteints de démence? Oui Non
15. • Avez-vous été vacciné contre le COVID-19?

- Oui, une dose
- Oui, deux doses
- Non

o Si oui, quel vaccin vous a-t-il administré
☐ Le vaccin AstraZeneca / Oxford
☐ Le vaccin BioNtech-Pfizer
☐ Le vaccin Moderna
☐ autre
o Si oui, date de vaccination (première seringue).

16. Souffrez-vous de l'une des conditions suivantes? Indiquez ce qui s'applique (options de réponses multiples).
    11. Maladie cardiaque et vasculaire
    12. Diabète
    13. Hypertension artérielle (hypertension)
    14. Maladie pulmonaire / cardiaque / rénale sévère
    15. Système immunitaire affaibli (troubles immunosuppresseurs) ou système immunitaire affaibli par l'utilisation à long terme de médicaments immunosuppresseurs (ex: chimiothérapie, patients transplantés, rhumatismes immunosuppresseurs, corticoïdes ...)
    16. Maladie cancéreuse active
    17. Aucune de ces réponses
17. Avez-vous été vacciné contre la grippe cette saison? (Cette question sera reflétée dans le questionnaire lors du dernier suivi)
18. Avez-vous déjà été testé pour le coronavirus (via PCR / test rapide AG / ou radiologique via CT scan)? Oui Non

19. Si oui, avez-vous déjà été testé positif pour le coronavirus? (Plusieurs options de réponse)
    - Oui, via un test PCR (test dans lequel un échantillon est prélevé via un écouvillon nasal et vous obtenez le résultat après analyse en laboratoire)

- Oui, via un test rapide d'antigène (test dans lequel un échantillon est prélevé via un tampon nasal et le résultat peut être lu en 15 minutes)
- Oui, radiologiquement, via un scanner
- non

14. Si oui, date du 1er test PCR positif ou test rapide (approximative)?
15. Si le COVID-19 a été confirmé radiologiquement par un scanner, indiquez la date de ce scanner.
16. Si vous avez déjà été testé positif, quelle était la source probable de contamination?
    - Au sein du WZC
    - En dehors du WZC
    - La source de la contamination m'était inconnue
17. Avez-vous ressenti un ou plusieurs des symptômes suivants pendant une période comprise entre février 2020 et maintenant, sans aucune explication claire à ce sujet? Indiquez ce qui s'applique (options de réponses multiples).
    - Toux
    - Essoufflement
    - Douleur thoracique
    - Perte de goût
    - Perte d'odeur
    - Aggravation des maladies respiratoires chroniques (ex: BPCO, asthme, toux chronique, ...)
    - Fièvre
    - fatigue musculaire
    - Fatigue
    - Nez qui coule / bouché
    - Gorge irritée
    - Mal de crâne
    - Perte d'appétit
    - La diarrhée
    - Confusion aiguë
    - Chute soudaine
18. Si oui, quand avez-vous ressenti ces symptômes? Veuillez indiquer le jour approximatif où ces symptômes ont commencé.

Version pour les résidents participants

(à remplir par le chercheur; à demander par l'infirmière (chef) ou le représentant du résident)

15. Veuillez saisir le numéro d'étudiant unique du participant. (liste cfr avec nombres aléatoires et numéros d'étude, retournée par le centre de recherche UGent)
16. Veuillez entrer l'âge du participant.
17. Quel est le sexe du participant? H / F
18. À quelle catégorie de soins appartient le résident?

O: A: B: Cc: CD: RÉ:

19. Le participant séjourne-t-il dans un service pour résidents atteints de démence?
20. • Le participant a-t-il été vacciné contre le COVID-19?

- Oui, une dose
- Oui, deux doses
- Non

o Si oui, quel vaccin le participant a-t-il reçu
 Le vaccin AstraZeneca / Oxford
 Le vaccin BioNtech-Pfizer
 Le vaccin Moderna
o Si oui, date de vaccination (première seringue).

21. Le participant souffre-t-il de l'une des conditions suivantes? Indiquez ce qui s'applique (options de réponses multiples).
    - Maladie cardiaque et vasculaire
    - Diabète
    - Hypertension artérielle (hypertension)
    - Maladie pulmonaire / cardiaque / rénale sévère
    - Système immunitaire affaibli (troubles immunosuppresseurs) ou système immunitaire affaibli par l'utilisation à long terme de médicaments immunosuppresseurs (ex: chimiothérapie, patients transplantés, rhumatismes immunosuppresseurs, ...)
    - Maladie cancéreuse active
    - Aucune de ces réponses
22. Le participant a-t-il déjà été testé pour le coronavirus (via PCR / test rapide AG / ou radiologiquement via CT scan)? Oui Non
23. Si c'est le cas. Le participant a-t-il déjà été testé positif pour le coronavirus?
    - Oui, via un test PCR (test dans lequel un échantillon est prélevé via un écouvillon nasal et vous obtenez le résultat après analyse en laboratoire)
    - Oui, via un test rapide d'antigène (test dans lequel un échantillon est prélevé via un tampon nasal et le résultat peut être lu en 15 minutes)
    - Oui, radiologiquement, via un scanner
    - Non
24. Si oui, date du 1er test PCR positif ou test rapide (approximative)?
25. Si le COVID-19 a été confirmé radiologiquement par un scanner, indiquez la date de ce scanner.
26. Le participant a-t-il ressenti un ou plusieurs des symptômes suivants pendant une période comprise entre février 2020 et maintenant, sans explication claire à ce sujet? Indiquez ce qui s'applique (options de réponses multiples).
    - Toux

- Essoufflement
- Douleur thoracique
- Perte de goût
- Perte d'odeur
- Aggravation des maladies respiratoires chroniques (ex: BPCO, asthme, toux chronique, ...)
- Fièvre
- fatigue musculaire
- Fatigue
- Nez qui coule / bouché
- Gorge irritée
- Mal de crâne
- Perte d'appétit
- La diarrhée
- Confusion aiguë
- Chute soudaine

27. Si oui, quand le participant a-t-il ressenti ces symptômes? Veuillez indiquer le jour où ces symptômes ont commencé.

QUESTIONNAIRE DE SUIVI

Questionnaire sur les foyers de soins résidentiels (à compléter tous les 2 mois dans la semaine où le test est administré)

1. Numéro de référence de l'étude du centre d'hébergement.

Les questions 2, 3, 4 ne sont ajoutées que si non dans le questionnaire précédent.

2. Une campagne de vaccination COVID a-t-elle déjà eu lieu au WZC?
3. . Date
    a. 1er tour / 2ème tour (date)
    b. Quel vaccin?
    i. Le Vaccin AstraZeneca / Oxford
    ii. Le vaccin BioNtech-Pfizer
    iii. Le vaccin Moderna
    iv. Autres
4. . Combien de résidents ont été vaccinés (par rapport au nombre total de résidents?)

. Combien d'employés ont été vaccinés (par rapport au nombre total d'employés?)

5.

6. Avez-vous eu des résidents / employés testés pour COVID-19 au cours des 2 derniers mois (entre le tour de test précédent et maintenant)?

7. Si c'est le cas,
    - Via le test PCR, combien?
    - Par le biais de tests antigéniques rapides, combien?

8. Si oui, qui vous a testé? Indiquez ce qui s'applique.
    - Résidents / employés présentant des symptômes
    - Un département / corridor (préventif, par exemple après contact avec le risque)
    - Tous les résidents / employés
    - Employés seulement
    - Résidents seulement
    - Autres combinaisons?: (Champ de texte ouvert)

9. Si oui, combien de résidents / employés ont été testés positifs?
    Pour le test PCR:
    Pour le test rapide:

10. Combien de résidents sont décédés du COVID-19 au cours des 2 derniers mois?
    - Nombre de décès COVID-19 confirmés
    - Nombre de décès suspects de COVID-19

Questionnaire individuel

(à compléter tous les 2 mois dans la semaine où le test est administré)

Version pour les employés participants:

18. Veuillez saisir votre numéro d'étudiant unique.
19. Participez-vous toujours à l'étude (ce questionnaire sera-t-il rempli)?
    Sinon, pourquoi pas?

À compléter par le chercheur

c. Absence provisoire de l'étude (maladie, vacances, hospitalisation ...)
d. Le participant ne souhaite plus participer
a. Mort suspectée ou prouvée de COVID-19 Date de décès:
b. Décès non liés au COVID-19 Date de décès:
c. L'employé ne travaille plus au WZC

La question 20 n'est ajoutée que si la réponse était non auparavant.

20. Avez-vous été vacciné contre le COVID-19?

- Oui, une dose
- Oui, deux doses
- Non

o Si oui, quel vaccin vous a-t-il administré
 Le vaccin AstraZeneca / Oxford
 Le vaccin BioNtech-Pfizer
 Le vaccin Moderna
 autre
o Si oui, date de vaccination (première seringue).

21.

22. Avez-vous été testé pour le coronavirus (via PCR / test rapide AG / ou radiologique via CT scan) au cours des deux derniers mois? Oui Non

23. Si c'est le cas. Avez-vous été testé positif au cours des 2 derniers mois (entre le cycle de test précédent et maintenant)? Plusieurs options de réponse
    - Oui, via un test PCR (test dans lequel un échantillon est prélevé via un écouvillon nasal et vous obtenez le résultat après analyse en laboratoire)
    - Oui, via un test rapide d'antigène (test dans lequel un échantillon est prélevé via un tampon nasal et le résultat peut être lu en 15 minutes)
    - Oui radiologiquement, via un scanner
    - non

24. Si oui, date du 1er test PCR positif ou test rapide au cours des 2 derniers mois (approximative)?
25. Si le COVID-19 a été confirmé radiologiquement par un scanner, indiquez la date de ce scanner.
26. Au cours des 2 derniers mois (entre la précédente série de tests et maintenant), avez-vous ressenti un ou plusieurs des symptômes suivants pendant une période, sans aucune explication claire? Indiquez ce qui s'applique (options de réponses multiples).
    - Toux
    - Essoufflement
    - Douleur thoracique
    - Perte de goût
    - Perte d'odeur
    - Aggravation des maladies respiratoires chroniques (ex: BPCO, asthme, toux chronique, ...)
    - Fièvre
    - fatigue musculaire
    - Fatigue
    - Nez qui coule / bouché
    - Gorge irritée
    - Mal de crâne
    - Perte d'appétit
    - La diarrhée
    - Confusion aiguë
    - Chute soudaine

27. Si oui, quand avez-vous ressenti ces symptômes? Veuillez indiquer le jour où ces symptômes ont commencé.

28. Le cas échéant, avez-vous été vacciné avec un vaccin COVID-19?
Dans l'affirmative, date de votre vaccination. (Cette question sera saisie dès que applicable)

Hésitation vaccinale au sein du personnel de soin des maisons de repos belges: questions additionnelles incluent une seule fois dans le questionnaire de suivi

A . Êtes-vous vacciné contre la Covid-19 ?

- Je suis déjà vacciné(e) (1 ou 2 doses).
  - Avez-vous hésité avant d'être vacciné(e) ? Oui/non
    - *Si oui → question B + C*
    - *Si non → fin de questionnaire*
- Je serai vacciné(e) prochainement.
  - Avez-vous hésité à vous faire vacciner ? Oui/non
    - *Si oui → question B + C*
    - *Si non → question D*
- J'hésite à être vacciné(e).
  - *Si oui → Question B*
- J'ai décidé de ne pas me faire vacciner.
  - *Si oui → Question E*

B . Quelle est la raison principale pour laquelle vous avez hésité à être vacciné contre la Covid-19 ?

- J'étais inquiet(e) à propos d'effets secondaires futurs inconnus.
- J'étais inquiet(e) des effets secondaires.
- Je n'avais pas confiance en la vaccination.
- J'avais l'impression d'avoir peu de chances d'être gravement malade à cause du coronavirus.
- Je pensais que l'impact du coronavirus est grandement exagéré.
- Je ne pensais pas que ça m'empêcherait d'attraper le coronavirus.
- Je présente une maladie qui rendrait le vaccin dangereux pour moi.
- J'ai déjà contracté le coronavirus donc je n'ai pas besoin du vaccin.
- J'ai peur des aiguilles.
- Je ne côtoie pas de personne à risque.
- Autre : _____(les participants peuvent donner une proposition)
- Je préfère ne pas répondre.

C . Quelle est la raison principale qui vous a fait changer d'avis à propos de la vaccination ?

- Je pense que les données des premières vaccinations étaient rassurantes.
- J’avais besoin de plus de temps pour m’informer.
- Un ami/membre de la famille/professionnel de la santé me l’a recommandé.
- J’ai été confronté(e) à une personne atteinte sévèrement du coronavirus.
- Un pourcentage de plus en plus grand de la population belge a accepté d’être vacciné.
- Je ressens le besoin de me faire vacciner pour protéger les personnes vulnérables.
- J’ai peur qu’une mesure m’interdise de voyager en n’ayant pas été vacciné(e).
- Je pense que le vaccin permettra de mettre fin à la crise sanitaire/économique.
- J’ai été soumis(e) à la pression d’un supérieur.
- J’ai été soumis(e) à la pression de mes proches.
- J’ai suivi les recommandations gouvernementales (conseils nationaux de sécurité, spots publicitaires, …).
- Je suis devenu(e) une personne à risque durant la crise.
- J’ai été informé(e) que j’étais une personne à risque de contracter une forme sévère du virus.
- J’ai été sensible à l’apparition de nouveaux variants du coronavirus plus contagieux.
- Autre : _____(*les participants peuvent donner une proposition*)
- Je préfère ne pas répondre

D . Quelle est la/les raison(s) du délai de vaccination ? (*les participants peuvent cocher plusieurs cases*)

- J’étais absent(e) du travail (vacances ou maladie) lors de la première vague de vaccination et je n’ai pas eu l’opportunité d’être vacciné(e) depuis.
- J’ai une maladie chronique et la vaccination ne m’était pas recommandée au moment de la première vague de vaccination.
- J’étais enceinte et la vaccination ne m’était pas recommandée au moment de la première vague de vaccination.
- J’envisage d’être enceinte prochainement.
- J’attendais un vaccin spécifique.
- Autre : _____ (*les participants peuvent donner une proposition*)
- Je préfère ne pas répondre.

E. Quelle est la raison principale pour laquelle vous ne voulez pas être vacciné(e) contre le coronavirus ?

- Je suis inquiet(e) à propos d’effets secondaires futurs inconnus.
- Je suis inquiet(e) des effets secondaires.
- Je n’ai pas confiance en la vaccination.
- J’ai l’impression d’avoir peu de chances d’être gravement malade à cause du coronavirus.
- Je pense que l’impact du coronavirus est grandement exagéré.
- Je ne pense pas que ça va m’empêcher d’attraper le coronavirus.
- Je présente une maladie qui rendrait le vaccin dangereux pour moi.

- J’ai déjà contracté le coronavirus donc je n’ai pas besoin du vaccin.
- J’ai peur des aiguilles.
- Je ne côtoie pas de personne à risque.
- Autre : _____(*les participants peuvent donner une proposition*)
- Je préfère ne pas répondre.

Version pour les résidents participants

(à remplir par l'examinateur; à demander par l'infirmière ou l'infirmière en chef ou le représentant du résident):

12. Veuillez saisir le numéro d'étude unique du participant.
13. Le résident participe-t-il toujours à l'étude (ce questionnaire sera-t-il rempli)? Oui Non
    Sinon, pourquoi pas?
    a. Décès COVID présumé ou prouvé (date du décès)
    b. Décès non lié au COVID-19 (date du décès)
    c. Le résident ne souhaite plus participer

La question 14 n'est ajoutée que si la réponse était non auparavant.

14. • Le participant a-t-il été vacciné contre le COVID-19?
    o Si oui, quel vaccin le participant a-t-il reçu
     Le vaccin AstraZeneca / Oxford
     Le vaccin BioNtech-Pfizer
     Le vaccin Moderna
    o Si oui, date de vaccination (première seringue).

15. Le participant a-t-il été testé pour le coronavirus (via PCR / test rapide ag / ou radiologiquement via CT scan) au cours des deux derniers mois? Oui Non
16. Si c'est le cas. Le participant a-t-il été testé positif pour le coronavirus au cours des 2 derniers mois (entre le cycle de test précédent et maintenant)?
    - Oui, via un test PCR (test dans lequel un échantillon est prélevé via un écouvillon nasal et vous obtenez le résultat après analyse en laboratoire)
    - Oui, via un test rapide d'antigène (test dans lequel un échantillon est prélevé via un tampon nasal et le résultat peut être lu en 15 minutes)
    - Oui, radiologiquement, via un scanner
    - Non

17. Si oui, date du 1er test PCR positif ou test rapide au cours des 2 derniers mois (approximative)?

18. Si le COVID-19 a été confirmé radiologiquement par un scanner, indiquez la date à laquelle cela s'est produit.

19. Le participant a-t-il ressenti un ou plusieurs des symptômes suivants au cours des 2 derniers mois (entre le cycle de test précédent et maintenant), sans aucune explication claire à ce sujet? Indiquez ce qui s'applique (options de réponses multiples).
    - Toux
    - Essoufflement
    - Douleur thoracique
    - Perte de goût
    - Perte d'odeur
    - Aggravation des maladies respiratoires chroniques (ex: BPCO, asthme, toux chronique, ...)
    - Fièvre
    - fatigue musculaire
    - Fatigue
    - Nez qui coule / bouché
    - Gorge irritée
    - Mal de crâne
    - Perte d'appétit
    - La diarrhée
    - Confusion aiguë
    - Chute soudaine

20. Si oui, quand le participant a-t-il ressenti ces symptômes? Veuillez indiquer le jour où ces symptômes ont commencé.

21. Le cas échéant, le participant a-t-il été vacciné avec un vaccin contre le COVID-19? Si oui, date de vaccination (Cette question sera inscrite dès que applicable)

6.4 Appendix 4: Sub-study: collection of Dry Blood Spot

Sub-study on (semi-)quantitative assessment of anti-SARS-CoV-2 specific antibodies upon COVID-19 vaccination

As part of the study on SARS-CoV-2 seroprevalence and seroconversion among residents and staff members of nursing homes in Belgium, a sub-study will be performed by Ghent University.

6.4.1 Objectives

(Semi)-quantitative follow-up of vaccine-induced and infection-induced anti-SARS-CoV-2 specific antibodies for assessment of COVID-19 vaccine effectiveness.

6.4.2 Study population

The study population will be the NH residents/ staff members tested for the purpose of the main seroprevalence study.

6.4.3. Study design

This sub-study follows the same time scheduling as the seroprevalence study. For each participant, in addition to every POC rapid antibody test, a few drops of capillary blood will be collected on a Dry Blood Spot saver card (4 saturated spots).

6.4.4. Data collection and analysis

Only biological specimens will be collected. No additional questionnaire will be needed. The collected dry blood spots will be send back to Ghent University and saved in a study-specific biobank. The collected samples will be analyzed using an Enzyme-Linked Immuno Sorbent Assay *(*ELISA*)* for (semi)-quantitative detection of anti-SARS-CoV-2 specific antibodies (anti-S1/anti-NCP). The Dry Blood Spot samples will be pseudonymized.

6.4.5 Risks

No additional risks.

6.4.6 Benefits

By longitudinal follow-up of the immune response in this study population, additional knowledge on COVID-19 vaccine effectiveness will be gained.

6.4.7. Informed consent and ethics

Permission for collection of the Dry Blood Spot will be asked in the informed consent form and ethical approval will be obtained for this sub-study by the ethical committee of University Hospital Ghent as part of the seroprevalence study.

References

[1] Roda WC, Varughese MB, Han D, Li MY. Why is it difficult to accurately predict the COVID-19 epidemic? Infect Dis Model 2020; 5: 271–81.

[2] Verity R, Okell LC, Dorigatti I, et al. Estimates of the severity of coronavirus disease 2019: a model-based analysis. Lancet Infect Dis 2020; 20: 669–77.

[3] Lipsitch M, Swerdlow DL, Finelli L. Defining the epidemiology of Covid-19-studies needed. N Engl J Med 2020; 382: 1194–96.

[4] WHO. Population-based age-stratified sero-epidemiological investigation protocol for COVID-19 virus infection. March 17, 2020. https://apps.who.int/iris/handle/10665/331656 (accessed May 18, 2020).

[5] https://www.thelancet.com/journals/lancet/article/PIIS0140-6736(20)31304-0/fulltext

[6] Rode Kruis, 2020. https://www.rodekruis.be/nieuws-kalender/nieuws/4-3-van-de-belgen-hebben-antistoffen-tegen-coronavirus-/

[7] Deborah Steensels, Els Oris, Laura Coninx, Dieter Nuyens, Marie-Luce Delforge, Pieter Vermeersch, Line Heylen. Hospital-Wide SARS-CoV-2 Antibody Screening in 3056 Staff in a Tertiary Center in Belgium. JAMA. 2020 Jun 15;e2011160. doi: 10.1001/jama.2020.11160.

[8] Laure Mortgat, Cyril Barbezange, Natalie Fischer, Leo Heyndrickx, Veronik Hutse, Isabelle Thomas, Bea Vuylsteke, Kevin Arien, Isabelle Desombere, Els Duysburgh. SARS-CoV-2 Prevalence and Seroprevalence among Healthcare Workers in Belgian Hospitals: Baseline Results of a Prospective Cohort Study. medRxiv 2020.10.03.20204545; doi: https://doi.org/10.1101/2020.10.03.20204545

[9]Sciensano. COVID-19 mortaliteit -update van de gegevens -26 augustus 2020. August 26, 2020. https://covid-19.sciensano.be/sites/default/files/Covid19/MORTALITEIT%20COVID-19%20%E2%80%93%20UPDATE%20VAN%20DE%20GEGEVENS%20-%2026%20AUGUSTUS%202020.pdf (accessed September 28, 2020).
