# Supplementary material for: Prevalence of SARS-CoV-2 antibodies among Belgian nursing home residents and staff during the primary COVID-19 vaccination campaign
Source: Eur J Gen Pract. 2022 Nov 28;29(2):2149732. doi: 10.1080/13814788.2022.2149732 (PMC10249443; doi:10.1080/13814788.2022.2149732)
Supplement: Table S3 [file IGEN_A_2149732_SM4491.docx]

**Table S3. Overview of the IgG/IgM rapid test results** **among residents and staff in Belgian nursing homes.**

|  | **Total study population**  **n=3,008** | | **Residents**  **n=1,640** | | **Staff**  **n=1,368** | |
| --- | --- | --- | --- | --- | --- | --- |
|  | n | **%** | n | **%** | n | **%** |
| **IgG/IgM rapid test results** | |  |  |  |  |  |
| No test | 23 | **1** | 11 | **1** | 12 | **1** |
| Invalid test | 24 | **1** | 5 | **0** | 19 | **1** |
| Negative | 770 | **26** | 503 | **31** | 267 | **20** |
| Positive |  |  |  |  |  |  |
| IgM | 21 | **1** | 12 | **1** | 9 | **1** |
| IgG | 1,285 | **43** | 580 | **35** | 705 | **52** |
| IgM+IgG | 885 | **29** | 529 | **32** | 356 | **26** |
| Self-sampled* | 185 | **6** | 30 | **2** | 155 | **11** |

*Sampling performed by nursing home medical staff.
