# Supplementary figures and images for: Prevalence of SARS-CoV-2 antibodies among Belgian nursing home residents and staff during the primary COVID-19 vaccination campaign
Source: Eur J Gen Pract. 2022 Nov 28;29(2):2149732. doi: 10.1080/13814788.2022.2149732 (PMC10249443; doi:10.1080/13814788.2022.2149732)

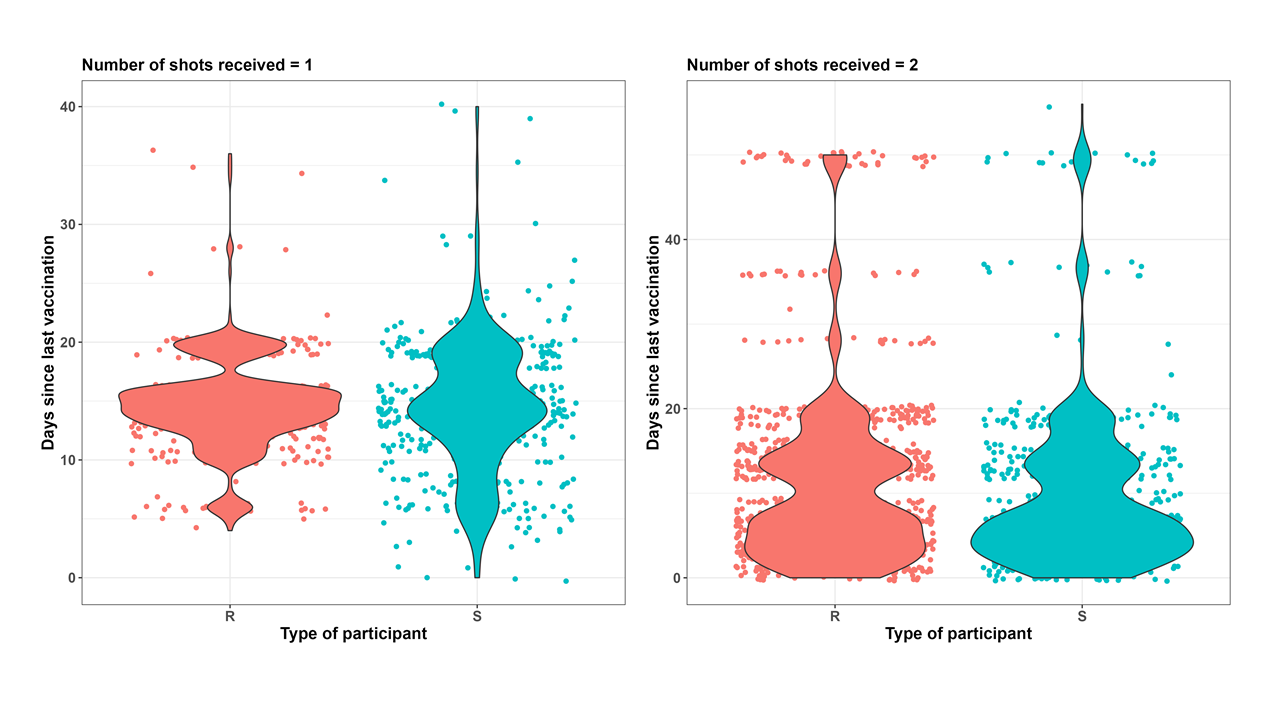

Supplement: Figure S1 [file IGEN_A_2149732_SM4490.tiff]
