# Supplementary material for: Prevalence of SARS-CoV-2 antibodies among Belgian nursing home residents and staff during the primary COVID-19 vaccination campaign
Source: Eur J Gen Pract. 2022 Nov 28;29(2):2149732. doi: 10.1080/13814788.2022.2149732 (PMC10249443; doi:10.1080/13814788.2022.2149732)
Supplement: Table S2 [file IGEN_A_2149732_SM4489.docx]

**Table S2. Distribution of the nursing homes, residents and staff in the study, and the total number of nursing homes, nursing home beds and nursing home staff in Belgium.**

|  | **Recruited NH in study** | | | | | |  | **Belgium** | | | | | |
| --- | --- | --- | --- | --- | --- | --- | --- | --- | --- | --- | --- | --- | --- |
|  | **Nursing homes** | | **Residents** | | **Staff** | |  | **Nursing homes** | | **Resident beds** | | **Staff** | |
|  | n | **%** | n | **%** | n | **%** |  | n | **%** | n | **%** | n | **%** |
| **Total** | 69 | **100** | 1,640 | **100** | 1,368 | **100** |  | 1,521 | **100** | 142,503 | **100** | 105,164 | **100** |
| **Region** |  |  |  |  |  |  |  |  |  |  |  |  |  |
| Brussels | 7 | **10** | 165 | **10** | 139 | **10** |  | 142 | **9** | 15,943 | **11** | 9,394 | **9** |
| Wallonia | 23 | **33** | 551 | **34** | 452 | **33** |  | 578 | **38** | 50,511 | **35** | 36,112 | **34** |
| Flanders | 39 | **57** | 924 | **56** | 777 | **57** |  | 801 | **53** | 76,049 | **53** | 59,658 | **57** |
| **Province** |  |  |  |  |  |  |  |  |  |  |  |  |  |
| Walloon Brabant | 3 | **4** | 71 | **4** | 60 | **4** |  | 52 | **3** | 4,751 | **3** | 3,400 | **3** |
| Flemish Brabant | 7 | **10** | 165 | **10** | 137 | **10** |  | 135 | **9** | 12,697 | **9** | 8,352 | **8** |
| Antwerp | 11 | **16** | 259 | **16** | 220 | **16** |  | 208 | **14** | 21,006 | **15** | 15,661 | **15** |
| Limburg | 5 | **7** | 120 | **7** | 100 | **7** |  | 99 | **7** | 8,851 | **6** | 7,501 | **7** |
| Liège | 7 | **10** | 168 | **10** | 133 | **10** |  | 179 | **12** | 15,644 | **11** | 11,298 | **11** |
| Namur | 3 | **4** | 72 | **4** | 59 | **4** |  | 75 | **5** | 6,152 | **4** | 4,517 | **4** |
| Hainaut | 8 | **12** | 192 | **12** | 160 | **12** |  | 230 | **15** | 20,510 | **14** | 14,273 | **14** |
| Luxembourg | 2 | **3** | 48 | **3** | 40 | **3** |  | 42 | **3** | 3,454 | **2** | 2,624 | **2** |
| West Flanders | 7 | **10** | 167 | **10** | 140 | **10** |  | 164 | **11** | 15,348 | **11** | 13,380 | **13** |
| East Flanders | 9 | **13** | 213 | **13** | 180 | **13** |  | 195 | **13** | 18,147 | **13** | 14,764 | **14** |
