# Supplementary material for: Prevalence of SARS-CoV-2 antibodies among Belgian nursing home residents and staff during the primary COVID-19 vaccination campaign
Source: Eur J Gen Pract. 2022 Nov 28;29(2):2149732. doi: 10.1080/13814788.2022.2149732 (PMC10249443; doi:10.1080/13814788.2022.2149732)
Supplement: Table S1 [file IGEN_A_2149732_SM4488.docx]

**Table S1. Overview of the definition of the different care-dependency levels according to the Katz evaluation scale (independence in activities of daily living) [10].**

| **Care level** | **Definition** |
| --- | --- |
| **O** | Independent |
| **A** | Physically dependent for washing and/or getting dressed.  OR  Psychologically dependent (disorientated in time and space, but physically independent) |
| **B** | Physically dependent:  - for washing and/or getting dressed  - for relocating/traveling and/or going to the toilet.  OR  Psychologically dependent (disorientated in time and space) and physically dependent for washing and/or getting dressed. |
| **C** | Physically dependent (but psychologically independent):  - for washing and/or getting dressed.  - for relocating/traveling and/or going to the toilet.  - Incontinent and/or need assistance with eating. |
| **Cd** | Physically dependent:  - for washing and/or getting dressed.  - for relocating/traveling and/or going to the toilet.  - Incontinent and/or need assistance with eating.  AND  Psychologically dependent (disorientated in time and space) and/or diagnosed with dementia based on a specialized diagnostic report,  performed by a physician-specialist in neurology, in psychiatry or geriatrics. |
| **D** | Diagnosed with dementia based on a specialized diagnostic report,  performed by a physician-specialist in neurology, in psychiatry or geriatrics. |
